# Lyophilized mRNA-lipid nanoparticle vaccines with long-term stability and high antigenicity against SARS-CoV-2

**DOI:** 10.1038/s41421-022-00517-9

**Published:** 2023-01-23

**Authors:** Liangxia Ai, Yafei Li, Li Zhou, Wenrong Yao, Hao Zhang, Zhaoyu Hu, Jinyu Han, Weijie Wang, Junmiao Wu, Pan Xu, Ruiyue Wang, Zhangyi Li, Zhouwang Li, Chengliang Wei, Jianqun Liang, Haobo Chen, Zhimiao Yang, Ming Guo, Zhixiang Huang, Xin Wang, Zhen Zhang, Wenjie Xiang, Dazheng Sun, Lianqiang Xu, Meiyan Huang, Bin Lv, Peiqi Peng, Shangfeng Zhang, Xuhao Ji, Huiyi Luo, Nanping Chen, Jianping Chen, Ke Lan, Yong Hu

**Affiliations:** 1 Shenzhen Rhegen Biotechnology Co. Ltd., Shenzhen, Guangdong China; 2 State Key Laboratory of Virology, College of Life Sciences, ABSL-3 Laboratory/Institute for Vaccine Research, TaiKang Center for Life and Medical Sciences, Wuhan University, Wuhan, Hubei China; 3 Jiangsu Rec-biotechnology Co. Ltd., Taizhou, Jiangsu China; 4 Wuhan Recogen Biotechnology Co. Ltd., Wuhan, Hubei China

**Keywords:** Immunology, Molecular biology

## Abstract

Advanced mRNA vaccines play vital roles against SARS-CoV-2. However, most current mRNA delivery platforms need to be stored at −20 °C or −70 °C due to their poor stability, which severely restricts their availability. Herein, we develop a lyophilization technique to prepare SARS-CoV-2 mRNA-lipid nanoparticle vaccines with long-term thermostability. The physiochemical properties and bioactivities of lyophilized vaccines showed no change at 25 °C over 6 months, and the lyophilized SARS-CoV-2 mRNA vaccines could elicit potent humoral and cellular immunity whether in mice, rabbits, or rhesus macaques. Furthermore, in the human trial, administration of lyophilized Omicron mRNA vaccine as a booster shot also engendered strong immunity without severe adverse events, where the titers of neutralizing antibodies against Omicron BA.1/BA.2/BA.4 were increased by at least 253-fold after a booster shot following two doses of the commercial inactivated vaccine, CoronaVac. This lyophilization platform overcomes the instability of mRNA vaccines without affecting their bioactivity and significantly improves their accessibility, particularly in remote regions.

## Introduction

After years of research and development, breakthroughs have been made in mRNA delivery systems, and mRNA vaccines have become frontrunners that preventing coronavirus disease 2019 (COVID-19). Compared with inactivated vaccines and recombinant protein vaccines, mRNA vaccines could be easily and quickly updated to target different variants with comparable first-in-class protective efficacy^1^. The relatively simple sequence-independent manufacturing process saved substantial time and cost in the development of a new vaccine, especially in pandemic conditions, with the emergence of new variants of severe acute respiratory syndrome coronavirus 2 (SARS-CoV-2)^2,3^.

Current mRNA therapeutics heavily depend on lipid or lipid-like delivery systems to improve theirs in vivo transfection efficacy^4^. Several components form lipid nanoparticles (LNPs) carrying mRNA to targeted organelles or tissues. Although mRNA therapeutics have advantages, challenges in physiochemical stability still impede their accessibility^5^. Cryogenic preservation and transportation are needed for the two current licensed mRNA vaccines, BNT162b2 (−80 °C to −60 °C) and mRNA-1273 (−20 °C)^6^. The stringent requirements come from the complex interactions among multiple lipid components and the instability of mRNA, which is sensitive to oxygen, moisture, enzymes, pH, and other conditions^7^. Recently, Packer et al.^8^ reported a novel mechanism by which the oxidation and subsequent hydrolysis by-products of ionizable lipids could accelerate the deterioration of mRNA. As water, oxygen, and ionizable lipids are common components in mRNA-LNP solutions, it is difficult to achieve high stability of the liquid mRNA-LNPs in principle. Zhao et al.^9^ reported an LNP vaccine that was stable at 2–8 °C for 6 months. Unfortunately, the study did not give details about the formulation or the transportation stability, as LNP solution usually does not tolerate vibration.

Lyophilization, a process that removes water by sublimation under a vacuum at a low temperature, seems to be a promising solution^10^. It is a relatively mild drying method applicable to vulnerable biomacromolecules or colloidal nanoparticles^11^. After the removal of water and oxygen, lyophilized mRNA could be stored at room temperature for a long time^12,13^. However, the drying of mRNA-LNPs is a more sophisticated technique, as the freezing and dehydration process introduces mechanical forces that deform the vehicle structure, leading to vehicle aggregation, mRNA breakage, or leakage^14^. Moreover, some researchers showed that even though mRNA-LNPs retained their integrity and encapsulation efficiency (EE), the in vivo transfection efficacy was greatly reduced after lyophilization due to unknown causes^15^. Muramatsu et al.^16^ reported a lyophilized mRNA-LNP with good thermostability at 4 °C and 25 °C for 24 weeks, while at 40 °C, the mRNA integrity was sharply decreased.

Here, we present an optimized lyophilization technique with precise temperature control and lower residual water content, which could effectively maintain the physiochemical properties and bioactivity of mRNA-LNPs during long-term storage at 2–8 °C, room temperature, and even 40 °C. The improved thermostability was verified with mRNA-LNPs containing different mRNA molecules, demonstrating the wide applicability of the technique.

Furthermore, we utilized this technique to prepare the first lyophilized, thermostable mRNA-LNP vaccines encoding the antigen (NTD–RBD region of the Spike protein) of SARS-CoV-2 wild-type (WA1, LyomRNA-WT), Delta (LyomRNA-Delta) or Omicron (LyomRNA-Omicron) viruses and confirmed their high-level antibody responses and immunogenicity.

Notably, we optimized the mRNA sequences through rational design by our proprietary algorithm in these lyophilized vaccines. The lyophilized SARS-CoV-2 vaccines exhibited strong immunogenicity in mice, rabbits, and old monkeys and elicited high titers of neutralizing antibodies and potent cellular immune responses. In humans, the administration of LyomRNA-Omicron as a booster shot showed excellent performance and could effectively increase the titers of neutralizing antibodies against wild-type coronavirus and different Omicron variants by at least 253-fold after a booster shot following two CoronaVac inactivated vaccines.

## Results

### Lyophilized mRNA-LNPs exhibited high thermostability and bioactivity

The four kinds of mRNA-LNPs used in this study were prepared through a classic T-junction mixing process and then lyophilized^17^. LNPs containing *Luciferase* mRNA (mRNA-Luc LNPs), Wild-type mRNA (mRNA-WT LNPs), Delta mRNA (mRNA-Delta LNPs), or Omicron mRNA (mRNA-Omicron LNPs) all showed a narrow size distribution and high EE, as listed in Supplementary Table S1. From the cryo-TEM images (Supplementary Fig. S1), we observed uniform, lamellar, and vesicular structures of the mRNA-LNPs.

Lyophilization of the LNPs was difficult due to the vulnerable nature of mRNA and the sophisticated structures and components in the nanoparticles. Here, we adopted an optimized lyophilization process to eliminate LNP damage during drying. During lyophilization, water molecules were removed upon sublimation and the sugar molecules were filled in to sustain the integrity of mRNA-LNPs^18^ (Supplementary Fig. S2a). After lyophilization, the dried mRNA-LNPs looked like a white fluffy cake (Supplementary Fig. S2b) and were readily and rapidly dissolved in water (< 10 s). The reconstituted solution was uniform and translucent, similar to freshly prepared mRNA-LNP solutions. The size, polydispersity index (PDI), and EE of mRNA-LNPs (Supplementary Table S1) changed only slightly, indicating that the optimized lyophilization process did not change their basic physical properties. The mRNA integrity (> 90%) and LNP integrity were also well maintained (Supplementary Fig. S3), demonstrating that the lyophilization process did not damage the mRNA structure.

**Fig. 1:**
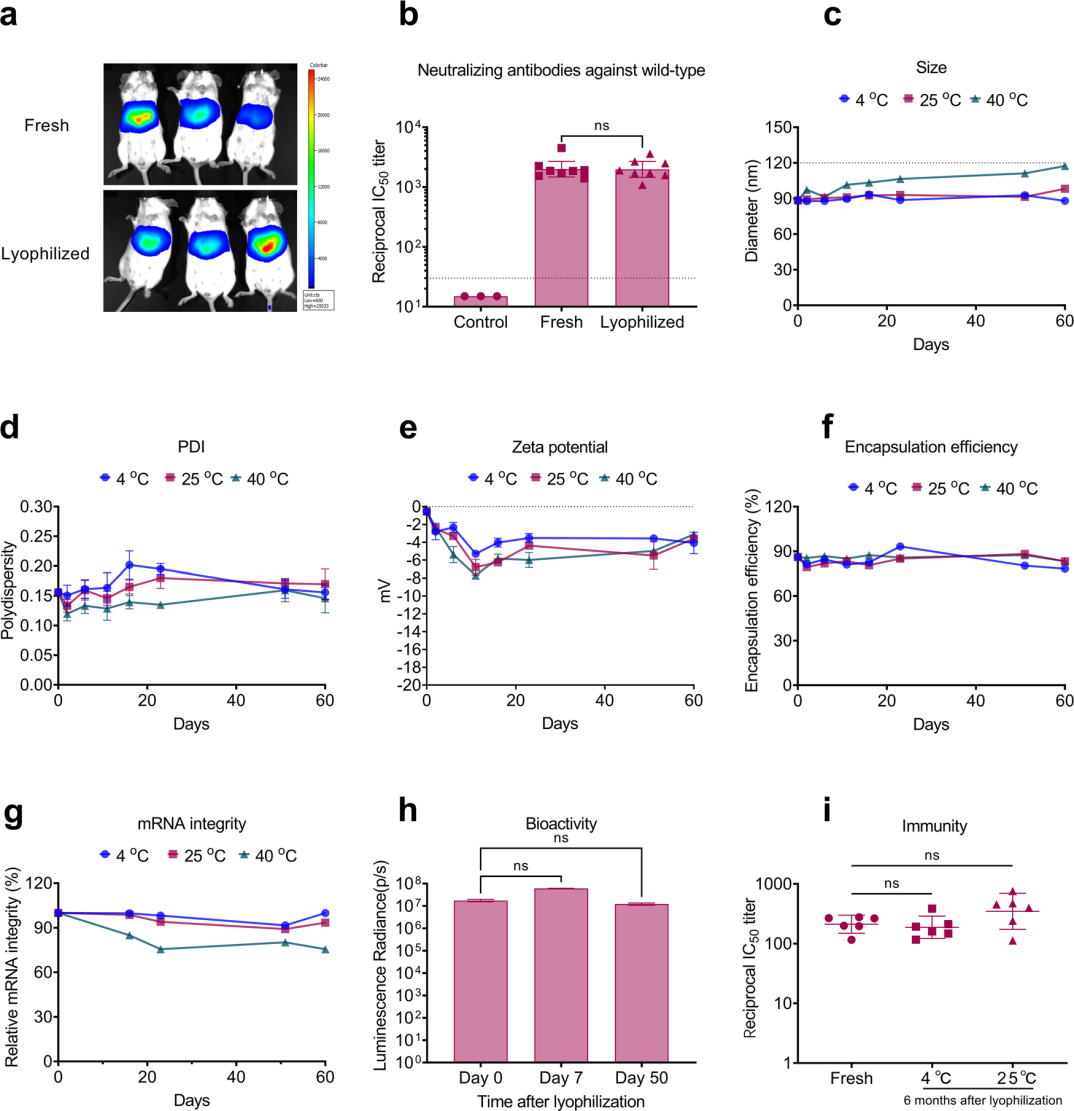
Lyophilized mRNA-LNPs exhibited potent bioactivity and thermostability. **a** Bioluminescent images of mice after intravenously injected with freshly prepared or lyophilized mRNA-Luc LNPs containing 5 μg mRNA. **b** Pseudotyped IC_50_ titer of mice after two doses with freshly prepared mRNA-WT LNPs or LyomRNA-WT LNPs. Mice (*n* = 8) were immunized with fresh or lyophilized mRNA-LNPs containing 5 μg mRNA at D0 and D7. Blood was collected and analyzed on D21. The dotted line represents the assay limit of detection. **c**–**g** Changes in the size (**c**), PDI (**d**), zeta potential (**e**), encapsulation efficiency (**f**), and mRNA integrity (**g**) of LyomRNA-Omicron LNPs after incubation at 4 °C, 25 °C, or 40 °C for 60 days. **h** Average luminescence radiance of mice (*n* = 3) after treatment with LyomRNA-Luc LNPs containing 5 μg mRNA that had been stored for an extended period. **i** Pseudotyped IC_50_ titer of mice after one shot with freshly prepared mRNA-Omicron LNPs or LyomRNA-Omicron LNPs. Mice (*n* = 6) were injected with saline or mRNA-LNPs (fresh or lyophilized product after being incubated at 4 °C or 25 °C for 6 months) containing 1 μg mRNA at D0. Blood was collected and analyzed at D14. The neutralizing titers of the saline group were under the detection limit (30) and were not shown. **b** The vaccine groups were compared by a two-sided Mann–Whitney test. **h**, **i** Time points were compared to D0 or freshly prepared mRNA-LNPs by Kruskal-Wallis ANOVA with Dunn’s multiple comparisons test. ns, not significant. Data are presented as the geometric means ± 95% confidence interval (**b**, **i**) or means ± SEM (**c**–**h**).

The in vivo transfection efficiency of lyophilized mRNA-LNPs was first evaluated with *Luciferase* mRNA. As seen in Fig. 1a, mice treated with lyophilized mRNA-Luc LNPs (LyomRNA-Luc LNPs, 1.98 × 10^7^ p/s) showed comparable total luminescence intensity to those treated with freshly prepared mRNA-Luc LNPs (2.67 × 10^7^ p/s). Next, we investigated the immunogenicity of mRNA-WT LNPs after lyophilization with an accelerated immunization procedure. Mice were immunized with mRNA-WT LNPs or LyomRNA-WT LNPs at day 0 (D0) and D7 through intramuscular injection, and at D21, the serum was collected. The neutralizing response was tested at this time point through a pseudotyped virus assay (Fig. 1b). There was no significant difference in the titers against wild type for the groups treated with 5 μg of mRNA-WT LNPs (geometric mean titers, GMTs, 1991) and 5 μg of LyomRNA-WT LNPs (GMTs, 1973). These data demonstrated that the lyophilization process did not affect the bioactivity or immunogenicity of the mRNA-LNPs.

Furthermore, we evaluated the thermostability of lyophilized mRNA-Omicron LNPs (LyomRNA-Omicron LNPs) by incubating them at 4 °C, 25 °C, or 40 °C for different periods of time (Fig. 1c–g; Supplementary Fig. S4). The products did not exhibit obvious changes in size, PDI, EE, or mRNA integrity at 4 °C and 25 °C within 6 months (Supplementary Fig. S4). Moreover, the nanomorphology was well-maintained as well at 25 °C after 6 months (Supplementary Fig. S1). In the high-temperature challenge experiment (40 °C), the size of lyophilized mRNA-LNPs increased slightly, and the relative mRNA integrity was maintained as high as 75.6% after incubation for 60 days. Furthermore, we analyzed the lipids through high-performance liquid chromatography with a charged aerosol detector (HPLC-CAD). As the results showed (Supplementary Fig. S5), there was no sign of lipid degradation. The well-maintained physical properties of lyophilized mRNA-LNPs should come from the ultralow water content (< 2%) and oxygen content (no air leakage detected) in the sealing bottles.

The bioactivity was then evaluated after long-term storage. The LyomRNA-Luc LNPs after storage at 4 °C for 50 days showed no difference from freshly prepared Luc LNPs in the in vivo bioluminescence experiment (Fig. 1h). More importantly, after incubation for 6 months at 4 °C or 25 °C, LyoOmicron-LNPs still showed excellent immunogenicity, which induced high pseudotyped IC_50_ titers (Fig. 1i) and IgG titers (Supplementary Fig. S4f) comparable to freshly prepared mRNA-Omicron LNPs.

These results indicated that mRNA LNPs maintained their physiochemical properties and bioactivity well after lyophilization, and the thermostability was greatly improved. It was estimated that the lyophilized mRNA-LNPs could be stored at 2–8 °C or room temperature for an extended period of time.

### The high immune response induced by lyophilized mRNA-Delta vaccine

Since 2021, more SARS-CoV-2 variants have continued to emerge, among which Delta and Omicron are the variants with the greatest impact. The fast mutation rate and highly contagious nature of these variants caused heavy casualties and decreased the prevention ability of exist vaccines^19,20^. Under these conditions, the fast-updatable mRNA vaccine shows its advantages. For better prevention of the epidemic disease, two mRNA vaccines were prepared to further evaluate their efficacy against Delta and Omicron variants based on the lyophilized mRNA-LNP formulation (LyomRNA-Delta and LyomRNA-Omicron).

**Fig. 2:**
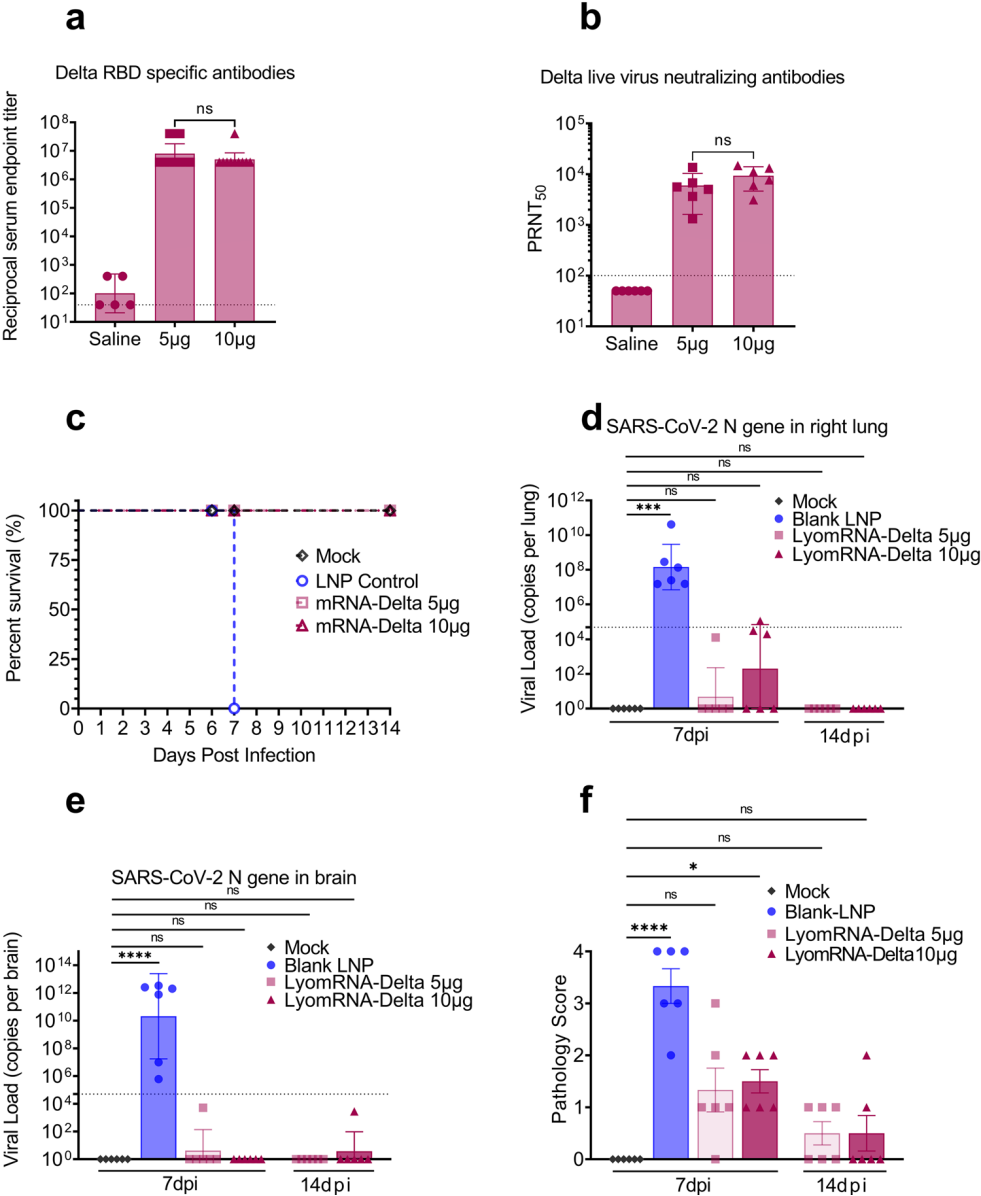
LyomRNA-Delta elicits a potent immune response and protects mice from infection by Delta viruses. **a** BALB/c mice (*n* = 10) were intramuscularly injected with saline or immunized twice at a dose of 5 μg or 10 μg at an interval of 14 days. Plasma samples were collected on D14 after the second dose to detect the Delta RBD-specific binding antibody titers. **b**–**f** K18-hACE2 transgenic mice were injected with saline, blank LNP (LNP without mRNA), or intramuscularly inoculated with 5 μg or 10 μg of LyomRNA-Delta vaccine twice at an interval of 14 days, and two weeks after the second dose, 2.5 × 10^3^ PFUs of SARS-CoV-2 Delta were administered to the animals intranasally. **b** Live SARS-CoV-2 Delta 50% neutralization titers 14 days after the second dose. **c** Animal survival curves after challenge. All blank LNP animals died or were euthanized 7 days after the challenge. **d**–**f** The virus load in the lungs (**d**), virus load in the brain (**e**), and pathology score (**f**) were evaluated 7 and 14 days after the challenge. Data are presented as the geometric means ± 95% confidence interval (**a**, **b**), geometric means ± geometric SD (**d**, **e**), or means ± SEM (**f**). **a**, **b** Doses were compared by a two-sided Mann–Whitney test. **f** Groups were compared to mock by Kruskal–Wallis ANOVA with Dunn’s multiple comparisons test. ns, not significant. *P < 0.05, ***P < 0.001. The horizontal dashed line marks the lower limit of detection (LLOD). Values below the LLOD were set to half the LLOD.

First, mice received two immunizations with LyomRNA-Delta at an interval of 14 days. Fourteen days after the second immunization, serum samples were obtained to measure the binding antibodies and neutralizing antibodies. The results showed that the GMTs of neutralizing antibodies of the 5 μg and 10 μg groups reached 7,980,000 and 5,040,000, respectively (Fig. 2a); and the GMTs of neutralizing antibodies against true viruses reached 5978 and 9323, respectively (Fig. 2b), indicating that LyomRNA-Delta elicited a very potent humoral immune response.

After receiving two doses of 5 μg and 10 μg of LyomRNA-Delta vaccine, K18-hACE2 transgenic mice were challenged with SARS-CoV-2 Delta infection. The group that received blank LNPs (LNPs without mRNA inside) was highly susceptible to SARS-CoV-2 Delta infection. Beginning at 2 dpi (days post infection), mice showed clinical symptoms, including lethargy, ruffled fur, arched back, and drowsiness. At 6 dpi, body weight reduction was observed in the blank LNP control mice, and ~20% weight loss was found at 7 dpi. Two mice died, and the remaining mice were moribund and were humanely euthanized according to ethical principles. In contrast, mice that received two doses of reconstituted LyomRNA-Delta showed no clinical abnormality, with no difference in body weight from the mock mice (Fig. 2c; Supplementary Fig. S6a). For these groups, six mice were euthanized at 7 dpi, and the remaining six mice were euthanized at 14 dpi. After euthanasia, the brain and right lung lobes were collected for viral load quantification, and the left lung lobes were fixed for histopathological evaluation. The results showed that lyophilized mRNA-Delta vaccination successfully protected the mice from SARS-CoV-2 Delta infection. The viral load in the right lung tissue of LyomRNA-Delta-vaccinated mice was below the detection limit at 7 dpi, while a high level of viral load was detected in the right lung tissue of mice in the blank LNP control group, with up to ~10^11^ copies/lung.

Importantly, the viral load in the right lung tissue was almost undetectable in all LyomRNA-Delta-vaccinated mice at 14 dpi (below the limit of detection) (Fig. 2d). The viral load in the brain tissues in the 5 μg groups displayed similar patterns as those in the lung tissues. Additionally, LyomRNA-Delta 10 μg showed a remarkable protective effect on virus dissemination and amplification (Fig. 2e).

Moreover, the pathological study showed that at 7 dpi, the vaccinated mice had markedly less severe lung tissue injuries than the blank LNP group, with no significant difference in the pathology score from the mock mice. At 14 dpi, the lung tissues of the vaccinated mice showed further improvement (7 dpi average pathology score: 5 μg dose group, 1.3; 10 μg dose group, 1.5; and 14 dpi average pathology score: 5 μg dose group, 0.5; 10 μg dose group, 0.5) (Fig. 2f; Supplementary Fig. S6b).

Together, these data suggest that the lyophilized mRNA-Delta vaccine was highly immunogenic and could fully protect challenged mice from SARS-CoV-2 Delta infection.

### Lyophilized Omicron mRNA vaccine could simultaneously elicit high levels of humoral and cellular immunity

**Fig. 3:**
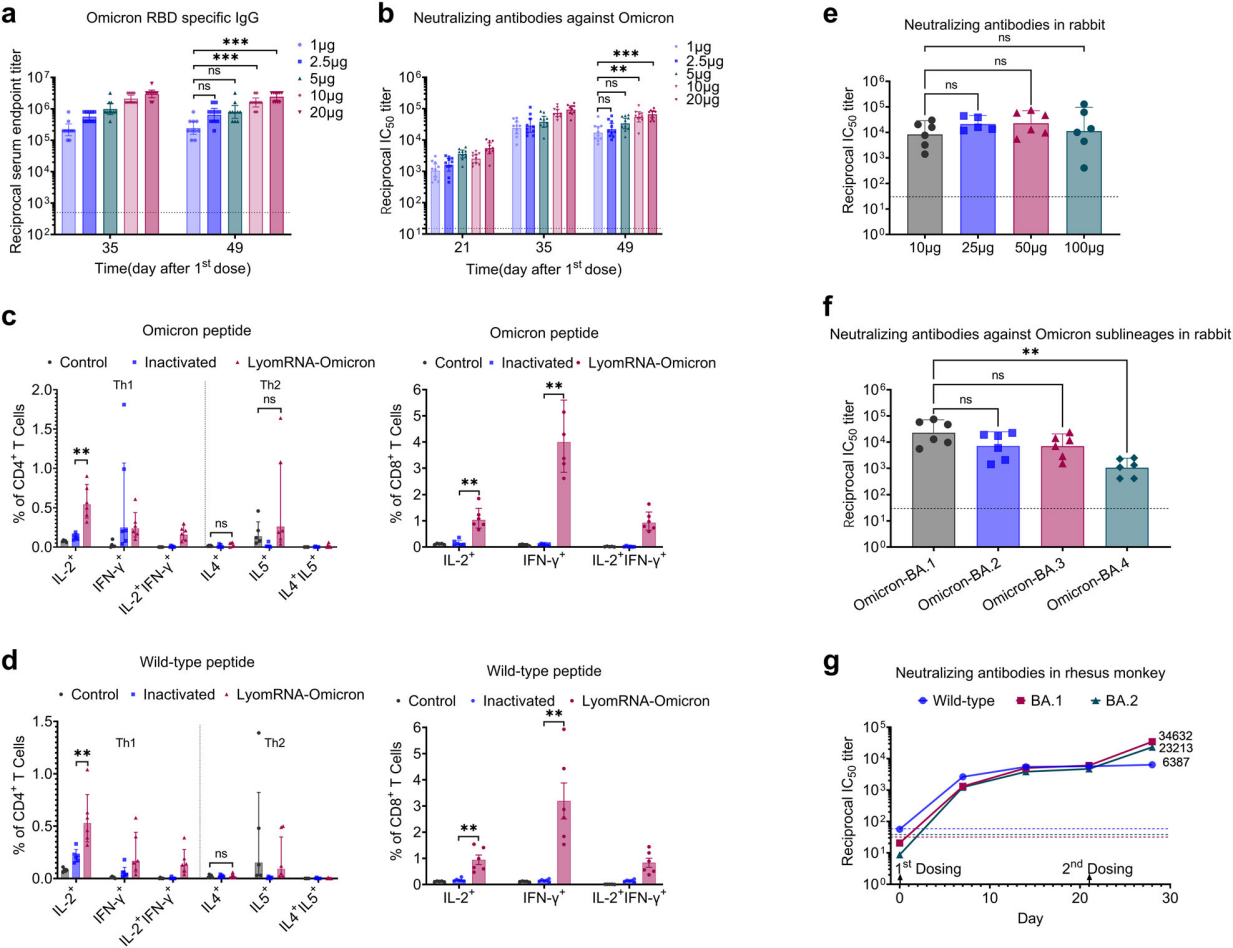
LyomRNA-Omicron could simultaneously activate high levels of humoral and cellular immunity. **a**, **b** C57BL/6N mice (*n* = 10) received two muscular injections of different doses of LyomRNA-Omicron. The time of the first dose was considered as D0, and the second dose was given at D21. Plasma Omicron RBD-specific IgG binding antibody titers (**a**) and titers of pseudovirus neutralizing antibodies at different time points following the first dose (**b**) are shown. **c**, **d** C57BL/6N mice were vaccinated with 5 μg of LyomRNA-Omicron or 0.65 U of the inactivated vaccine at an interval of 21 days. The spleens were obtained 28 days after the second shot for ICS. The control mice received normal saline. The frequencies of IL2/IFN-γ/IL4/IL5-positive CD4^+^ T cells and IL2/IFN-γ-positive CD8^+^ killer cells specific for SARS-CoV-2 Omicron NTD-RBD peptide (**c**) or wild-type NTD–RBD peptide (**d**) were detected. **e**, **f** New Zealand rabbits, received two muscular injections of vaccines at an interval of 21 days. The plasma-neutralizing antibodies for pseudoviruses (**e**) and pseudoviruses of all Omicron variants at a dose of 50 μg (**f**) were detected 14 days after the last immunization. **g** Six-year-old monkeys were immunized twice with 50 μg of LyomRNA-Omicron (*n* = 3) at a 3-week interval. Blood samples were collected before and after vaccination. Neutralizing antibodies against the wild-type, Omicron variant BA.1 and BA.2 were tested by a VSV-pseudovirus-based system. Immunogenicity data were collected at 7, 14, 21, and 28 days after the first dose of LyomRNA-Omicron. **a**, **b**, **e**, **f** Group comparisons were made by Kruskal–Wallis ANOVA with Dunn’s multiple comparisons test. **c**, **d** Vaccine groups were compared by a two-sided Mann–Whitney test. ns, not significant. **P < 0.01, ***P < 0.001. Data are presented as the geometric means ± 95% confidence interval (**a**, **b**, **e**, **f**, **g**) or geometric means ± geometric SD (**c**, **d**). Dotted lines represent assay limits of detection for **a**, **b**, **e**, **f** and plasma pseudovirus neutralizing antibody titers of control monkeys without immunization for **g**.

The Omicron variant has become the predominant SARS-CoV-2 strain worldwide. Consequently, we specifically developed the freeze-dried mRNA vaccine, LyomRNA-Omicron, based on the sequence of the Omicron variant. LyomRNA-Omicron-induced neutralizing antibody levels are comparable to those induced by the freshly prepared mRNA-Omicron vaccine (Supplementary Fig. S7a). In mice, a scheme consisting of two immunizations at an interval of 21 days led to a positive antibody conversion rate of 100% after the first shot of LyomRNA-Omicron. At D21, the GMT of neutralizing antibodies reached 1064 in the 1 μg dose group. The titer of neutralizing antibodies continued to climb after the second immunization and was 23-fold higher at D35 than at D21, with the GMT reaching 24,256. Meanwhile, the titers of neutralizing antibodies correlated positively with the dosage; however, there was no significant difference in the dose range from 1 µg to 5 µg. In addition, the titer of binding antibodies for the Omicron RBD showed an identical trend (Fig. 3a, b). Furthermore, in the binding antibody typing experiment, the LyomRNA-Omicron-activated Omicron-specific RBD IgG2a/IgG ratio was greater than 1 (Supplementary Fig. S8a).

We also examined LyomRNA-Omicron cellular immunity by intracellular cytokine staining (ICS). The results showed that in C57 and Balb/c mice stimulated by the Omicron NTD–RBD peptide pool, LyomRNA-Omicron could effectively activate IL2^+^ and IFN-γ^+^ Th1 CD4^+^ cells, and the ratio of activated CD4^+^ cells was higher than that with the inactivated vaccine (CoronaVac, inactivated whole wild-type virus vaccine by Sinovac Life Sciences). Moreover, there was no significant difference in activated IL4^+^ and IL5^+^ Th2 CD4^+^ cells compared with the PBS control group. LyomRNA-Omicron also activated the production of massive amounts of CD8^+^ killer T cells against Omicron, and the ratio of activated cells was also significantly higher than that of the inactivated vaccine group (Fig. 3c; Supplementary Figs. S8b, S9a, S10a–c). Intriguingly, LyomRNA-Omicron could also effectively activate the cellular immune response against SARS-CoV-2 wild-type viruses (Fig. 3d; Supplementary Fig. S9b).

Next, we examined the immune response to LyomRNA-Omicron in rabbits. Two-dose immunization with an interval of 21 days was used, and the levels of neutralizing antibodies were detected at D35. The results showed that the GMT reached 8337 with 10 μg of LyomRNA-Omicron, and there was no significant difference in antibody levels in the dose range from 10 µg to 100 μg (Fig. 3e). Moreover, LyomRNA-Omicron could effectively neutralize Omicron BA.1/BA.2/BA.3 subvariants. Although it had certain neutralizing activities against BA.4, the GMT was reduced by 20-fold compared to that for BA.1 (Fig. 3f).

The elderly population is most vulnerable to SARS-CoV-2. Therefore, we used 6-year-old monkeys to evaluate the level of immunity against LyomRNA-Omicron. In the primary immunization experiment with two-dose immunization at an interval of 21 days, 50 μg LyomRNA-Omicron simultaneously elicited the production of neutralizing antibodies against Omicron-BA.1, Omicron-BA.2 and the wild-type virus in old monkeys (Fig. 3g).

In summary, LyomRNA-Omicron could elicit effective humoral immunity and Th1-dominant cellular immunity.

### Lyophilized Omicron mRNA vaccine could offer immune protection as a booster shot in animals

Multiple key mutations in Omicron allow the viruses to escape the protection of primary immunization with vaccines that were designed based on the sequence of the original wild-type virus^21^. Therefore, booster immunity is critical for warding off Omicron. To date, at least two-thirds of the world’s population has received at least one shot of the SARS-CoV-2 vaccine, and inactivated vaccine is one of the most commonly administered vaccines, especially in China^22^. Therefore, we designed a heterogeneous booster experiment with LyomRNA-Omicron based on inactivated vaccine CoronaVac.

**Fig. 4:**
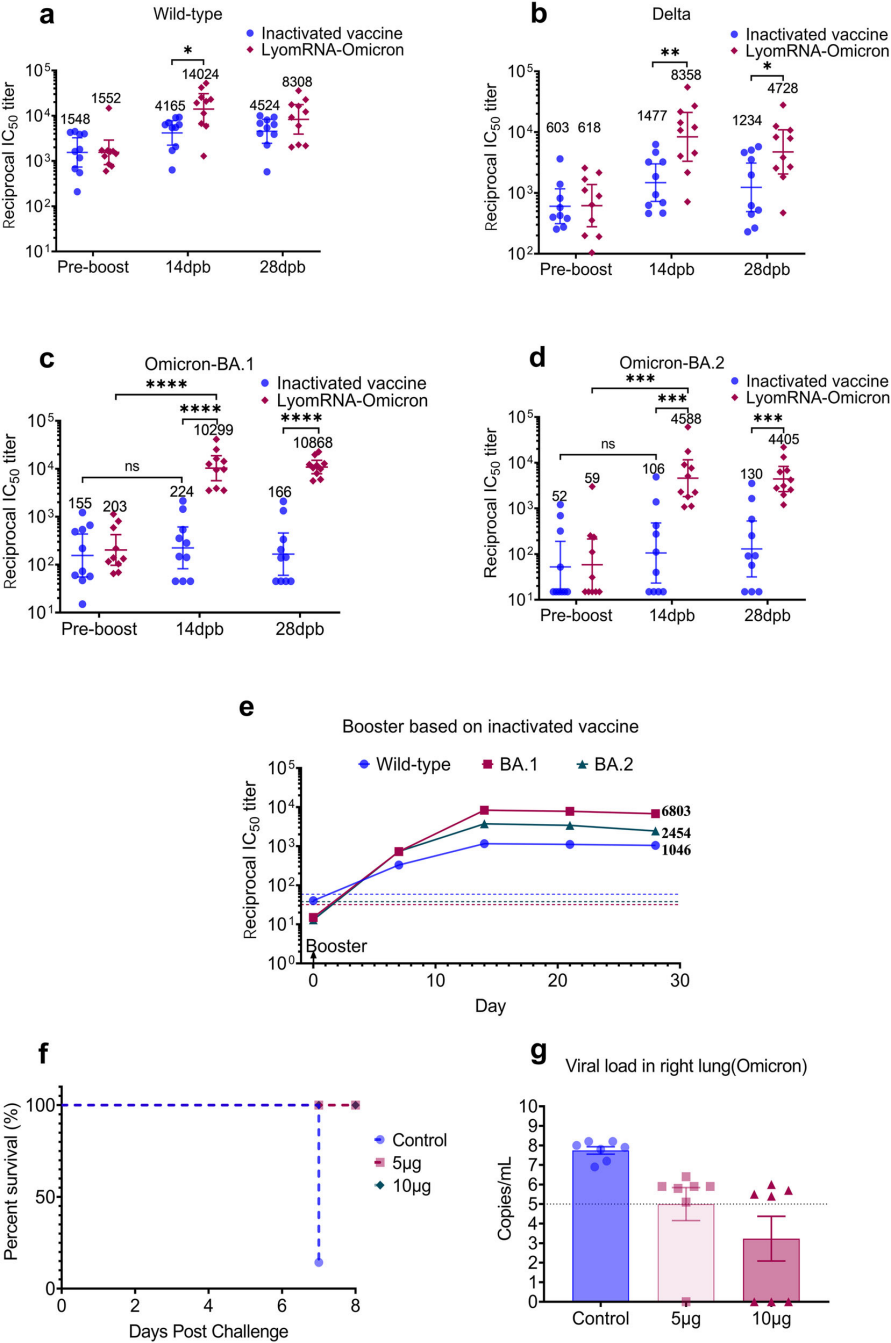
LyomRNA-Omicron booster immunization engendered significant immune protection. **a**–**d** BALB/c mice (*n* = 10) were immunized with the inactivated vaccine (0.65 U per dose) at D0 and D21, and a booster shot with heterogeneous LyomRNA-Omicron (5 μg) or homogeneous inactivated vaccine (0.65 U) was given at D42. The titers of plasma neutralizing antibodies against wild-type (**a**), Delta (**b**), Omicron-BA.1 (**c**), and Omicron-BA.2 (**d**) pseudoviruses were detected pre-boost, or at 14 days post boosting (dpb) and 28 dpb. **e** Six-year-old monkeys that had received two doses of inactivated vaccines (6.5 U dose per animal, twice at a 3-week interval) were boosted 18 months later with 50 μg LyomRNA-Omicron. Blood samples were collected before or 7, 14, 21, and 28 days after boosting with LyomRNA-Omicron. Neutralizing antibodies against the wild-type viruses, Omicron variants BA.1 and BA.2 were tested by a VSV-pseudovirus-based system. Dotted lines represent the blood pseudovirus-neutralizing antibody titers of nonimmunized control monkeys. **f**, **g** K18-hACE2 transgenic mice were vaccinated at D0, D21, and D61 with LyomRNA-Omicron (5 μg dose), and were lethally challenged at D73 with Omicron-BA.1. Survival (**f**) and lung virus load on D7 post challenge (**g**) were recorded. **a**–**d** The groups were compared by a two-sided Mann–Whitney test. *P < 0.05, **P < 0.01, ***P < 0.001, ****P < 0.0001. Data are presented as the geometric means ± 95% confidence interval (**a**–**e**) or means ± SEM (**g**).

In mice, the third dose with LyomRNA-Omicron after two immunization doses with CoronaVac elicited high levels of neutralizing antibodies against the wild-type, Delta, Omicron-BA.1, and Omicron-BA.2 variants simultaneously, and the GMT was increased by 9-, 13.5-, 50.7-, and 77.7-fold over that before vaccination, respectively. The titer was significantly higher than that elicited by a third booster shot with homogeneous CoronaVac and was 3.4, 5.7, 46, and 43.3 times higher than the latter, respectively (Fig. 4a–d).

In 6-year-old monkeys, a heterogeneous booster with LyomRNA-Omicron 18 months after primary immunizations with two-dose CoronaVac could also elicit the production of high levels of neutralizing antibodies against the wild-type, Omicron-BA.1 and Omicron-BA.2, with GMTs of 1046, 6803, and 2454, respectively (Fig. 4e), indicating that although the levels of neutralizing antibodies against SARS-CoV-2 were low in primates long after immunization with inactivated vaccine, a booster with LyomRNA-Omicron could still induce high levels of neutralizing antibodies.

In addition, we designed a homogeneous booster experiment with LyomRNA-Omicron in hACE2 transgenic mice. Two primary shots were performed at D0 and D21, followed by the third booster shot at D73. Mice were lethally challenged with Omicron-BA.1. The results showed that the survival rate of mice immunized with a low (5 μg) or high (10 μg) dose reached 100%, and 6/7 nonimmunized control mice died on D7 post challenge (Fig. 4f). The viral load of the right lung and pathological section of the left lung revealed that compared to nonimmunized mice, the immunized mice were clearly protected against Omicron (Fig. 4g; Supplementary Fig. S11).

The data demonstrated that in animal studies, the use of LyomRNA-Omicron as a booster showed remarkable immunization effects.

### Lyophilized Omicron mRNA vaccine booster immunization experiment in humans

Most people in China have received inactivated SARS-CoV-2 vaccines. Booster immunization is a feasible approach to prevent breakthrough infection by Omicron. We tested the immunization effect of LyomRNA-Omicron as a booster shot in humans. Before the investigator-initiated human trial, we performed in-use stability analysis by incubating the reconstituted vaccines at 4 °C or room temperature, which showed no change in immunogenicity within 8 h (Supplementary Fig. S7b, c). The high stability in use would facilitate clinical application.

A total of 26 volunteers participated in the experiment (Supplementary Table S2). 19 of them had received 2 doses of inactivated vaccines, and it has been more than 6 months since the last shot. They were assigned to cohort I, that is, a heterogeneous booster with LyomRNA-Omicron based on the previous two-dose immunization with inactivated vaccines. The remaining 7 participants had received 3 doses of inactivated vaccines and it has been at least 2 months since the last shot. They were assigned to cohort II, that is, a heterogeneous booster with LyomRNA-Omicron based on previous three-dose immunizations with inactivated vaccines.

**Fig. 5:**
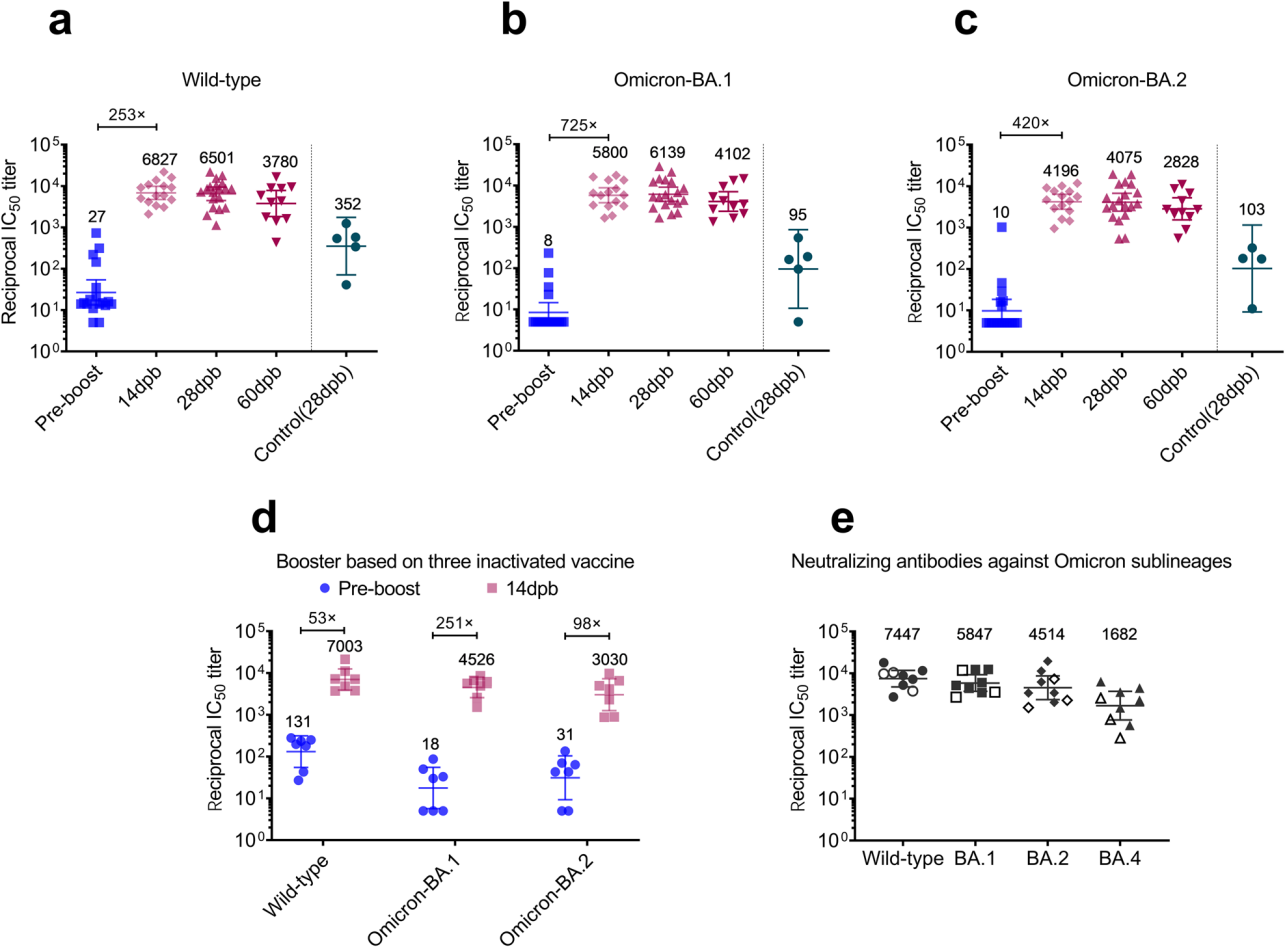
Booster immunization with LyomRNA-Omicron could elicit high titers of neutralizing antibodies in humans. **a**–**c** On the basis of two vaccinations of inactivated vaccine (at least 6 months after the second dose), a booster shot with LyomRNA-Omicron was administered. Then, the titers of neutralizing antibodies against the wild-type (**a**), Omicron-BA.1 (**b**), and Omicron-BA.2 (**c**) pseudoviruses were detected at D0 (pre-boost), 14 dpb, and 60 dpb. Plasma pseudovirus-neutralizing antibodies in the control group were detected one month after the third homogeneous booster shot with inactivated vaccine. **d** A fourth booster with LyomRNA-Omicron was given on the basis of three immunizations with inactivated vaccine (at least 2 months after the final dose), and then the titers of plasma-neutralizing antibodies against the wild-type, Omicron-BA.1, and Omicron-BA.2 pseudoviruses at 14 dpb were detected. **e** The titers of plasma neutralizing antibodies against Omicron 14 days after LyomRNA-Omicron booster immunization (nine serum samples were selected randomly for pseudovirus neutralization assay of Omicron, three of which boosting on three-dose inactivated vaccine are shown as empty dots, and six of which boosting on two-dose inactivated vaccine are shown as filled dots). Data are presented as the geometric means ± 95% confidence interval.

In cohort I, the pre-booster plasma samples of the subjects contained very low levels of neutralizing antibodies, with a GMT of 27 against the wild-type strain, and the titer was nearly 0 for Omicron-BA.1 and Omicron-BA.2. Fourteen days after boosting with 50 μg of LyomRNA-Omicron, the GMTs of neutralizing antibodies against the wild-type, Omicron-BA.1, and Omicron-BA.2 all significantly elevated, reaching 6827, 5800, and 4196, respectively, and increased by 253-, 725- and 420-fold. The high levels of antibodies were maintained at 1 and 2 months post boost. Among them, the GMT of antibodies at D28 after heterogeneous boosting with LyomRNA-Omicron was significantly higher than that of the control group (using the inactivated vaccine as the third shot) and was 18.5-, 64.6-, and 39.6-fold higher for the wild-type, Omicron-BA.1, and Omicron-BA.2 compared with the latter (Fig. 5a–c; Supplementary Fig. S12a).

In cohort II, LyomRNA-Omicron was used as the fourth booster shot, and the GMTs of neutralizing antibodies at D14 after the booster shot against the wild type, Omicron-BA.1, and Omicron-BA.2 were 7003, 4526, and 3030, respectively, and were increased by 131-, 251-, and 98-fold compared with those before the booster shot. Interestingly, the titers of neutralizing antibodies were comparable for the heterogeneous booster with LyomRNA-Omicron after two or three inoculations with inactivated vaccines (Fig. 5d; Supplementary Fig. S12b).

Currently, Omicron-BA.4/BA.5 are more infectious than the wild-type virus and are spreading quickly. We found that in humans, receiving booster immunization with LyomRNA-Omicron on the basis of inactivated vaccines, the GMT of neutralizing antibodies against BA.4 was reduced by a mild 4.4-fold compared with that against the wild type and 3.5-fold compared with that for BA.1, with the latter consistent with previous reports^23,24^. Interestingly, this is different from the 20-fold steep reduction for BA.4 compared to BA.1 in rabbits, reflecting immune differences between humans and animals. However, the levels of neutralizing antibodies against BA.4 were still high after boosting with LyomRNA-Omicron, with the GMT reaching 1682 (Fig. 5e).

In addition, booster immunization with LyomRNA-Omicron elicited a potent cellular immune response to the Omicron variants (Supplementary Fig. S13).

No grade 2 adverse events were observed in the 26 volunteers who received LyomRNA-Omicron, and no one had a body temperature above 37.5 °C. Four persons showed mild symptoms, including redness, and swelling in the muscular injection site in 1 subject, dizziness in 1 subject, and pain in the axillary lymph nodes in 2 subjects, all of which disappeared within 5 days.

In summary, LyomRNA-Omicron is suitable as a booster shot based on inactivated vaccines and could elicit excellent immune effects on the SARS-CoV-2 wild-type and Omicron variants and, at the same time, has mild side effects.

## Discussion

The mRNA-LNP vaccine has several advantages, such as rapid antigen design, the potential for quick, inexpensive, and scalable manufacturing, and the induction of strong humoral and cellular immunity, which make it a powerful weapon against infectious diseases, especially against those caused by viruses that are easy to mutate. The advantage and efficacy of mRNA-LNP vaccines have also been proven by two approved and marketed vaccines against COVID-19, Comirnaty, and Spikevax.

According to the data in Our World in Data as of 11th July 2022, 66.8% of the world population has received at least one dose of a COVID-19 vaccine. In total, 12.15 billion doses have been administered globally. However, only 20% of the population in low-income countries have received at least one dose of vaccine^22^. This is mainly because of the instability of mRNA-LNP vaccines, which require ultracold temperatures (−80 °C to −60 °C) for storage and transportation, greatly limiting their accessibility. Therefore, it is particularly important to develop an mRNA-LNP vaccine that can be transported in conventional cold chains for broad application.

In this study, we successfully achieved the long-term storage of an mRNA-LNP vaccine through lyophilization. The optimized lyophilization process preserved the nanostructures and physiochemical properties of mRNA-LNP and maintained their bioactivity as well, showing no significant difference from the freshly prepared vaccines. Moreover, LyomRNA-Omicron exhibited much-improved thermostability at 4 °C and 25 °C, without obvious deterioration after incubation for 6 months, or only slight deterioration at 40 °C after 2 months (stability data not shown for LyomRNA-WT vaccine and LyomRNA-Delta vaccine).

The lyophilized mRNA-WT, mRNA-Delta, and mRNA-Omicron vaccines could all produce high levels of IgG or neutralizing antibodies. In the challenge study of the LyomRNA-Delta vaccine against the Delta strain, it was confirmed that the vaccine could fully protect mice from infection and clear the virus. In the two-dose primary vaccination scheme, LyomRNA-Omicron elicited an immune response by Th1-biased CD4^+^ T cells and CD8^+^ killer T cells against both the Omicron and wild-type strains. In addition, it induced a wide-ranging and high-level immune response against wild-type, Delta, and Omicron variants as a booster for inactivated vaccines both in mice and old monkeys, with better efficacy than the inactivated vaccines. In human studies, LyomRNA-Omicron as a booster shot on the basis of inactivated vaccines caused fewer side effects, without a single case of fever. Following the booster shot, the production of neutralizing antibodies against Omicron can be elicited 100%, and the titers of neutralizing antibodies against the wild-type, Omicron-BA.1 and BA.2 strains were increased by at least 253-fold compared with those before the booster shot. Furthermore, toward the rapidly spreading Omicron-BA.4, the titers of neutralizing antibodies only decreased 3.5-fold compared to those against BA.1 but remained at an elevated level.

When LyomRNA-Omicron is subjected to an advanced mRNA-LNP freeze-drying process, which makes it more thermostable and accessible, it enters the ranks of advanced and excellent vaccines, solving the storage and transportation issues of current mRNA-LNP vaccines.

In conclusion, the lyophilized mRNA-LNP vaccine has the advantage of both high immunogenicity and accessibility and is very suitable for the prevention of epidemics such as SARS-CoV-2.

## Materials and methods

### Ethics statement

Mouse experiments were conducted by certified staff at the Center for Animal Experiments of Wuhan University and approved by the Institutional Animal Care and Use Committee (AUP #WP2021-0607). Female BALB/c mice aged 6–8 weeks and heterozygous *B6/JGpt-H11*^*em1Cin(K18-ACE2)*^*/Gpt* mice (K18-hACE2 KI transgenic mice) aged 5–7 weeks and weighing 16–18 g were used. The mice were housed with a 12-h dark–light cycle at a constant temperature (22 °C ± 2 °C, 45%–65% relative humidity) under pathogen-free conditions.

New Zealand rabbits weighing 2–2.5 kg (half male and half female) were purchased from Beijing Longan Experimental Animal Breeding Center. Relative experiments were conducted at Abzymo Biosciences Co., Ltd., which was accredited by Beijing Municipal Science and Technology Commission (SCXK (BJ) 2019-0006).

A nonhuman primate study was performed at Hunan Key Laboratory of Pharmacodynamics and Safety Evaluation of New Drugs.

The protocols and procedures of experiments using infectious SARS-CoV-2 viruses under the Animal Biosafety Level-III Laboratory facility were approved by the Institutional Biosafety Committee (IBC, Protocol #S01322010B). All samples were inactivated according to IBC-approved standard procedures for the removal of specimens from high containment.

For investigator-initiated human trials, the protocol and informed consent were approved by Wuhan University and Life Medical Ethics Committee at Wuhan University (IRB2022003). Written informed consent was obtained from the participants before immunization. This study was conducted in accordance with the Declaration of Helsinki and Good Clinical Practice.

### Materials

Lipids were purchased from Xiamen Sinopeg Biotech Co., Ltd. Cholesterol was purchased from AVT (Shanghai) Pharmaceutical Tech Co., Ltd. Ethanol was obtained from Aladdin. Citric acid and trisodium citrate were obtained from Sigma‒Aldrich. SARS-CoV-2 wild-type, Delta and Omicron pseudoviruses constructed using a recombinant replication-deficient vesicular stomatitis virus (VSV) vector that encodes luciferase instead of VSV-G (VSVΔG-Luc) were obtained from Gobond Testing Technology (Beijing) Co., Ltd., except for those in Supplementary Fig. S12, which were purchased from Vazyme Biotech Co., Ltd. (Nanjing). The Omicron NTD–RBD peptide pool (15mers with 11 aa overlap) was synthesized by GL Biochem Co., Ltd. (Shanghai).

### RNA synthesis

The SARS-CoV-2 NTD–RBD region was used as an antigen in this work. The relative amino acid sequences are shown below. Wild-type: amino acids 1–541 from the N-terminus of the Spike protein of Wuhan-Hu-1. Delta: amino acids with mutations T19R, V70Fa, T95I, G142D, E156–, F157–, R158G, A222Va, W258La, K417Na, L452R, T478K of wild type. Omicron: amino acids with mutations A67V, D69–70, T95I, G142D, D143–145, D211, L212I, +214EPE, G339D, S371L, S373P, S375F, K417N, N440K, G446S, S477N, T478K, E484A, Q493R, G496S, Q498R, N501Y, Y505H of wild type.

The respective N1-methylpseudouridine-modified mRNAs were produced in vitro by a standard T7 RNA polymerase-mediated transcription reaction, added to Cap1, and then purified through fast protein liquid chromatography. *Luciferase* mRNA was synthesized using the same method and purified by the MEGAclear™ Transcription Clean-Up Kit (Invitrogen).

### LNP preparation and characterization

The mRNA-loaded LNPs (mRNA-LNPs) were prepared by mixing an aqueous phase containing mRNA with an ethanol phase containing lipid mixtures using a T-junction mixing device as reported previously^17^. In brief, mRNA was dissolved in citrate buffer (100 mM, pH 4.0). The lipid mixtures were dissolved in anhydrous ethanol at a molar ratio of 46.3:9.4:42.7:1.6 for ionizable lipid, 1,2-distearoyl-sn-glycero-3-phosphocholine (DSPC), cholesterol, and PEG-lipid. The N/P ratio was maintained at 6:1. Then, the ethanol and aqueous phases were mixed at a volume ratio of 3:1 in the T-junction device. Thereafter, mRNA-LNPs were dialyzed against a buffer at pH 7.4 for 6 h. Then, mRNA-LNPs were sterilized with a 0.22-μm filter and stored at 4 °C for further use. The average diameter, PDI and zeta potential were measured with an NS-90Z (Malvern Panalytical). The concentration of leaked mRNA (*C*_leak_) was determined with a fluorescence detection kit following the manufacturer’s protocols. In addition, mRNA-LNPs were lysed with 0.4% Triton X-100 to determine the concentration of total mRNA (*C*_total_). The EE was calculated using the following equation:

$${{{\mathrm{Encapsulation}}}}\;{{{\mathrm{efficiency}}}}\;({{{\mathrm{EE}}}}) = \frac{{{{{{C}}}}_{{{{\mathrm{total}}}}} - {{{{C}}}}_{{{{\mathrm{leak}}}}}}}{{{{{{C}}}}_{{{{\mathrm{total}}}}}}} \times 100{{{\mathrm{\% }}}}$$

### Lyophilization and stability analysis

The mRNA-LNP solution was added to the cryoprotectant and placed in a penicillin bottle, and then the mixture was frozen at −40 °C and lyophilized with a freeze dryer (Pilot-2H, Boyikang). The temperature was increased to 25 °C gradually within 40 h. The resulting powder was collected, characterized, and stored at 4 °C for further use. The water content was measured through coulomb titration following the manufactures’ instructions (Mettler, C10S). The air leakage was tested by vacuum attenuation following the manufactures’ instruction (Sumspring, Leak-S). To measure the stability, lyophilized mRNA-LNPs were incubated at 4 °C, 25 °C, or 40 °C (40% humidity) at different times. LNP integrity was measured with a gel retardation assay as previously reported^25^. The mRNA integrity was tested with microfluidic capillary electrophoresis (Agilent Fragment Analyzer)^26^, and relative mRNA integrity was calculated as the percentage relative to the mRNA-LNPs before freeze drying (94% integrity). The lipid composition was analyzed with HPLC-CAD (Thermo Vanquish) with a C18 column (Acclaim^TM^, 300 Å, 2.1 × 150 mm) using water (0.5% TEAA) and methanol (0.5% TEAA) as mobile phases.

### Cryo-TEM

The cryo-TEM images were obtained from the Southern University of Science and Technology. In brief, grids (Quantifoil R 1.2/1.3 300 mesh) were first glow discharged for 60 s at 15 mA with a Pelco easiGlow glow discharge unit, and 4 μL of LNP suspension was applied to the surface of each grid. The grids were then blotted with filter paper (Whatman, Grade 1) for 5.5 s, blot force 0, and after 10 s, plunge-frozen in liquid ethane using a Vitrobot Mark IV. The sample chamber of the Vitrobot had a relative humidity of 95% and a temperature of 4 °C. The grids were imaged using a 300 kV Titan Krios electron microscope (Thermo Fisher Scientific) equipped with a GIF-Quantum energy filter (Gatan), and a slit width of 20 eV was used. Images were recorded with a Gatan K2 direct electron detector operating in super-resolution counting mode at a pixel size of 2.14 Å, with a dose rate of 15 electrons per pixel per second and a total exposure time of 15 s.

### Cell culture

Vero E6 (ATCC), HEK 293T/17 (ATCC), and ACE2-expressing 293T cells (Zhejiang Meisen Cell Technology Co., Ltd.) were cultured in Dulbecco’s modified Eagle’s medium supplemented with 2 mM l-glutamine, 10% FBS (Sigma-Aldrich) and 1% penicillin/streptomycin (BI) at 37 °C with 5% CO_2_.

### Luciferase transfection in vivo

Luciferase mRNA-loaded LNPs (mRNA-Luc LNPs) and their lyophilized products, LyomRNA-Luc LNPs, were examined for their in vivo transfection efficiency. The mRNA-Luc LNP solution (0.5 mL) containing 0.1 mg/mL encapsulated mRNA was freeze-dried in a penicillin bottle. Lyophilized LNPs were reconstituted using 0.5 mL of nuclease-free water. Mice were treated with 50 μL of mRNA-LNP solution through intravenous injection into the tail vein. At 24 h after injection, the mice were narcotized with avertin solution (0.2%, 350 μL). Then, the luciferin solution (1.4%, 200 μL) was administered intraperitoneally. The mice were imaged with an IVIS Spectrum in vivo imaging system (FluoView400, Boluteng).

### Immunization

Three kinds of mRNA-LNPs with mRNA encoding the antigen of wild-type (LyomRNA-WT), Delta (LyomRNA-Delta) or Omicron (LyomRNA-Omicron) SARS-CoV-2 variants were prepared and used for mouse immunization experiments. The mRNA-LNP solution (0.5 mL) containing 0.1 mg/mL encapsulated mRNA was freeze-dried in penicillin bottles. Lyophilized mRNA-LNPs were reconstituted using 0.5 mL nuclease-free water.

Mice received different doses of freshly prepared or reconstituted LyomRNA-LNPs through intramuscular injections. The immunization procedures and blood collection time intervals varied in different experiments, as indicated in the Results and Discussion.

New Zealand rabbits were immunized twice with different doses (10 μg, 25 μg, 50 μg, 100 μg) of LyomRNA-Omicron through intramuscular injection at a 21-day interval. Fourteen days after the last shot, neutralizing antibody titers were detected after blood collection.

Six-year-old male rhesus macaques were immunized on D0 and D21 with 50 μg of LyomRNA-Omicron for the primary vaccination. Phlebotomy was conducted on D0, D7, D14, D21, and D28, followed by serum-neutralizing antibody detection. For the booster scheme, 18 months after two doses of SARS-CoV-2 inactivated vaccine immunization, 50 μg of LyomRNA-Omicron was administered as a booster. Serum-neutralizing antibodies were analyzed before or 7, 14, 21, and 28 days after boosting.

Twenty-six people were involved in the clinical study. Nineteen of them have been vaccinated with inactivated vaccine twice (Cohort I) and the rest of them have received three doses of inactivated vaccine (Cohort II). In this study, they were immunized with 50 μg of LyomRNA-Omicron through deltoid intramuscular injection. The blood was collected before boosting, or 14, 28, and 60 days after boosting. The sex, age, and other information were listed in Supplementary Table S2.

The blood samples above were collected and centrifuged at 1500× *g* and 4 °C for 10 min. The supernatant sera were separated, aliquoted, and frozen at −80 °C before use.

### Enzyme-linked immunosorbent assay (ELISA)

Ninety-six-well ELISA microplates (Greiner) were coated with 2 ng/µL RBD protein (Novoprotein) in coating buffer (Dakewe) at 4 °C for 15 h. After washing and blocking, serially diluted mouse sera were incubated in plates at 4 °C for 2 h. After washing again, the secondary antibody, goat anti-mouse IgG H&L-conjugated HRP (Abcam), was incubated in plates at room temperature for 1 h. Then, 3′5′-tetramethylbenzidine (TMB) (Dakewe) was used as the substrate to detect antibody responses. Data were collected using a microplate reader (Molecular Devices) and SoftMax Pro software version 7.1.0. Endpoint titers were calculated as the dilution at which the absorbance exceeded 4 times that the background (secondary antibody alone).

### Pseudotyped virus neutralization assay

Neutralizing antibody titers were tested as reported^27^. Briefly, serum samples were diluted and mixed with 1.3 × 10^4^ TCID50/mL pseudotyped virus and incubated at 37 °C and 5% CO_2_ for 30 min. Thereafter, 293T-ACE2 cells were added and incubated for 24 h. Then, the Bio-Lite luciferase detection reagent (Vazyme) was mixed and incubated for 2 min. The luminescence values (RLU) were immediately detected with a microplate reader, and the amount of pseudotyped virus entering cells was calculated by detecting the expression of luciferase to obtain the neutralizing antibody content of the sample. Luminescence readout data were collected, and the half-maximal inhibitory concentration (IC_50_) was calculated for the tested samples.

### Authentic virus neutralization assay

SARS-CoV-2 Delta-neutralizing antibody was determined by in vitro inhibition of the cytopathic effect (CPE) and was performed in the Animal Biosafety Level 3 Laboratory at the Center of Laboratory Animal Sciences, Wuhan University (Wuhan, China). Sera from K18-hACE2 KI mice were serially diluted 3-fold in cell culture medium from 1:100 to 1:218,700. The diluted samples were mixed with a SARS-CoV-2 Delta virus suspension of 100 plaque-forming units (PFUs), followed by 1 h of incubation. The virus-serum mixtures were added to Vero-E6 cells seeded in 24-well plates and cultured in a 5% CO_2_ incubator at 37 °C for 3 days. The inhibitory capacity of each sample dilution was assessed for CPE, and the neutralization titer was calculated as the reciprocal of the serum dilution required for 50% neutralization of viral infection.

### Intracellular cytokine staining

For ICS assays, mice were sacrificed 3 weeks after the second immunization, and the spleens were harvested. Spleen single-cell suspensions were prepared in lymphocyte isolation solution (Dakewe) by mashing tissue against the surface of a 70-µm cell strainer (Falcon). Erythrocytes were removed by density gradient centrifugation. Cells from each mouse were resuspended in R10 medium (RPMI 1640 supplemented with 10% HI-FBS, 1% Pen–Strep solution, 2 mM l-Alanyl-Glutamine, and HEPES), and cell viability was detected by a Countess 3 Automated Cell Counter (Invitrogen). For mouse ICS in T cells, 4 × 10^6^ spleen cells were incubated for 7 h at 37 °C in a 5% carbon dioxide incubator with a protein transport inhibitor cocktail (4 A Biotech) under two conditions: no peptide stimulation (DMSO) and stimulation with Omicron-BA.1 NTD–RBD peptide pools. Peptide pools were used at a final concentration of 4 µg/mL for each peptide. After stimulation, the cells were washed twice with PBS buffer and stained with a Zombie Aqua™ Fixable Viability kit (Biolegend, Cat# 423101) for 20 min at room temperature. Cells were then washed in cell staining buffer (BD, Cat# 554657) and resuspended in Fc Block (Biolegend, Cat# 101320) for 5 min on ice prior to immunostaining with a surface stain cocktail containing the following antibodies: I-A/I-E Alexa Fluor 488 (Biolegend, Cat# 107616), CD8a PerCP/Cyanine5.5 (Biolegend, Cat# 100734), CD44 Alexa Fluor 700 (Biolegend, Cat# 103026), CD62L BV650 (Biolegend, Cat#104453), CD4 BV605 (Biolegend, Cat# 100548), and CD3ε APC/Fire 750 (Biolegend, Cat# 152308) in cell staining buffer (BD). After 30 min of incubation, cells were washed twice and incubated with 500 µL of BD Fixation and Permeabilization Solution (BD, Cat# 554722, 1×) at 4 °C for 20 min, washed twice with BD perm wash buffer (BD, Cat# 554723), and stained with a cocktail of fluorescently labeled anti-cytokine antibodies: IFN-γ PE/Dazzle 594 (Biolegend, Cat# 505845), TNF-α PE/Cyanine7 (Biolegend, Cat# 506324), IL-2 BV421 (Biolegend, Cat# 503826), IL-4 PE (Biolegend, Cat# 504104), IL-5 APC (Biolegend, Cat# 504306), and CD3ε APC/Fire 750 (Biolegend, Cat# 152308) in cell staining buffer (BD). After 30 min, the cells were washed once with 1× perm wash buffer (BD) and resuspended in 200 µL of cell staining buffer (BD) prior to flow cytometric analysis. The fluorescent signals were analyzed by a CytoFLEX LX flow cytometer (Beckman) and FlowJo software (Tree Star, Inc.). Background cytokine responses to the no peptide condition were subtracted from those measured in the peptide pool for each individual mouse. Statistical comparison between groups was performed using GraphPad Prism 9 software.

### ELISpot assays

IFN-γ ELISpot assays of mice were performed with a Mouse IFN-γ Precoated ELISpot Kit according to the manufacturer’s instructions (Dakewe). A total of 1 × 10^5^ splenocytes per well were restimulated ex vivo with Omicron-BA.1 NTD–RBD peptide mix (2 μg/mL per peptide), negative control (DMSO), or positive control (50 ng/mL PMA + 1 μg/mL Innomycin), respectively.

IFN-γ ELISpot assays of 5 × 10^5^ human PBMCs per well were performed with a Human IFN-γ Precoated ELISpot Kit purchased from Dakewe. Cells were restimulated ex vivo with Omicron-BA.1 NTD–RBD peptide mix (4 μg/mL per peptide), negative control (DMSO), or positive control (2.5 µg/mL PHA), respectively.

Twenty-four hours after restimulation, biotinylated antibody and streptavidin-HRP were added successively after cell lysis. Then, AEC peroxidase substrate was added, and spots were counted using an ELISpot plate reader (Mabtech IRIS™ Fluorospot Reader, Mabtech). Spot numbers were evaluated using Mabtech Apex™ software v.1.1.52.121.

### SARS-CoV-2 Delta and Omicron challenge in K18-hACE2 KI transgenic mice

SARS-CoV-2 Delta or Omicron challenge was performed in K18-hACE2 KI transgenic mice at the Animal Biosafety Level 3 Laboratory, Wuhan University.

For the Delta variant challenge, thirty-six 6-week-old female K18-hACE2 KI transgenic mice were divided into the negative control group (*n* = 6), the blank LNP control group (*n* = 6), the low-dose vaccine group (*n* = 12, 5 μg/dose) and the high-dose vaccine group (*n* = 12, 10 μg/dose). The latter three groups were intramuscularly immunized with 0.1 mL of blank LNPs, 5 μg of LyomRNA-Delta, or 10 μg of LyomRNA-Delta at D0 and D14. On D28 (14 days after the second immunization), mice immunized with LyomRNA-Delta or only blank LNP were intranasally challenged with 2.5 × 10^3^ PFUs of SARS-CoV-2 Delta viruses. The challenged mice were observed for clinical symptoms and death and weighed on the day of infection and daily thereafter for up to 14 dpi. Mice from the blank LNP control group at 7 dpi, six mice from each vaccination group at 7 dpi, and six mice from each vaccination group at 14 dpi were sacrificed under anesthesia. Lung and brain tissues were collected to determine virus titers by qPCR and pathology scores by hematoxylin and eosin (H&E) staining.

For the Omicron challenge, thirty-six 6-week-old male K18-hACE2 KI transgenic mice were divided into the control group (*n* = 7), the low-dose vaccine group (*n* = 8, 5 μg/dose) and the high-dose vaccine group (*n* = 8, 10 μg/dose). The control group was injected with water. The low-dose and high-dose groups were intramuscularly immunized with 5 μg of LyomRNA-Omicron and 10 μg of LyomRNA-Omicron, respectively, on D0, D21, and D61. On D73, the control and vaccination groups were intranasally challenged with 1 × 10^5^ PFUs of SARS-CoV-2 Omicron-BA.1 viruses. The challenged mice were observed for clinical symptoms and death and weighed on the day of infection and daily thereafter. At 7 dpi, all the mice were sacrificed under anesthesia, followed by virus titer and pathological detection in the lung and brain tissues. RT-PCR assay for SARS-CoV-2 was conducted with the viral nucleic acid detection kit according to the manufacturer’s protocol (Shanghai BioGerm Medical Technology, Co., Ltd.). The detection limit of the real-time PCR reaction is 100 copies per reaction, which is scaled to a limit of detection of 5 × 10^4^ copies per lung or brain.

### Statistical analysis

Data were collected from at least six independent experiments for in vivo experiments. All other experiments were performed at least three times independently unless otherwise specified. Statistical analysis was mainly processed using Prism software version 9.0.0 (GraphPad Software Inc., San Diego, CA) and analyzed by Kruskal‒Wallis ANOVA with Dunn’s multiple comparisons test or two-sided Mann‒Whitney test. Differences were considered statistically significant when *P* < 0.05.

For pathology score evaluation, injuries such as bleeding and edema of the entire left lung tissue can be observed by H&E staining under low microscope magnification (10×), the evaluation method is to observe injury distribution from 6 independent visual fields and score them according to the following criteria (injury area/total area):

0 points: no damage;

1 point: mild injury, < 25% mild influx of inflammatory cells with cuffing around vessels;

2 points: moderate injury, increased inflammation with ∼25%–50% of the total lung involved;

3 points: severe inflammation involving 50%–75% of the lung;

4 points: almost all lung tissue contained inflammatory infiltrates.

## Supplementary information

Supplementary Information
