# Supplementary material for: Lyophilized mRNA-lipid nanoparticle vaccines with long-term stability and high antigenicity against SARS-CoV-2
Source: Cell Discov. 2023 Jan 23;9:9. doi: 10.1038/s41421-022-00517-9 (PMC9868121; doi:10.1038/s41421-022-00517-9)
Supplement: Supplementary file 1 — Supplementary Information [file 41421_2022_517_MOESM1_ESM.pdf]

Supplementary Information

Full title:

Lyophilized mRNA-lipid nanoparticle vaccines with long-term stability and high antigenicity against SARS-CoV-2

Running title:

Thermostable lyophilized mRNA vaccines against SARS-CoV-2

Liangxia Ai[1,†], Yafei Li[1,†], Li Zhou[2,†], Wenrong Yao[3], Hao Zhang[1], Zhaoyu Hu[1], Jinyu Han[1], Weijie Wang[1], Junmiao Wu[1], Pan Xu[1], Ruiyue Wang[1], Zhangyi Li[1], Zhouwang Li[1], Chengliang Wei[1], Jianqun Liang[1], Haobo Chen[1], Zhimiao Yang[1], Ming Guo[2], Zhixiang Huang[2], Xin Wang[2], Zhen Zhang[2], Wenjie Xiang[2], Dazheng Sun[1], Lianqiang Xu[1], Meiyan Huang[1], Bin Lv[1], Peiqi Peng[1], Shangfeng Zhang[1], Xuhao Ji[1], Huiyi Luo[1], Nanping Chen[1], Jianping Chen[3,4,*], Ke Lan[2,*], Yong Hu[1,4,*]

[1] Shenzhen Rhegen Biotechnology Co. Ltd, Shenzhen, Guangdong, China

[2] State Key Laboratory of Virology, College of Life Sciences, ABSL-3 Laboratory/Institute for Vaccine Research, TaiKang Center for Life and Medical Sciences, Wuhan University, Wuhan, Hubei, China

[3] Jiangsu Rec-biotechnology Co. Ltd, Taizhou, Jiangsu, China

[4] Wuhan Recogen Biotechnology Co. Ltd, Wuhan, Hubei, China

† These three authors contributed equally to this work.

*Corresponding author: Yong Hu (Email: yong.hu@rhegen.com)

Ke Lan (Email: klan@whu.edu.cn)

Jianping Chen (Email: chenjp@recbio.cn)

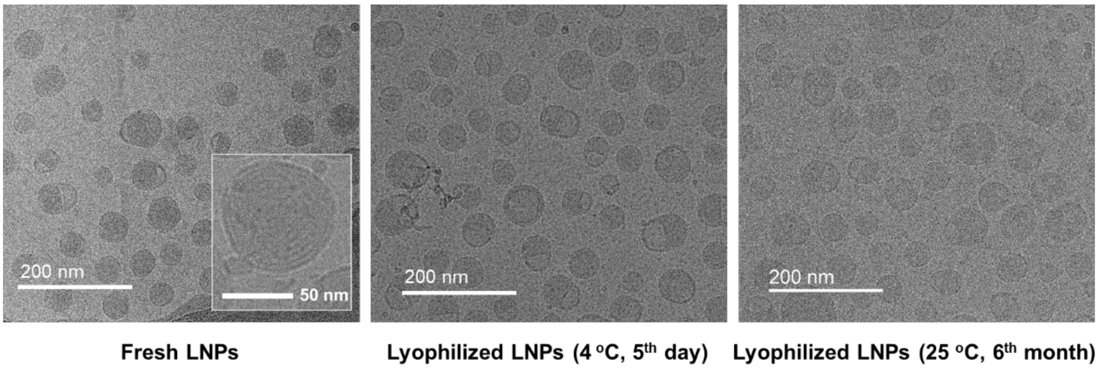

Supplementary Fig. S1| Cryo-TEM image of fresh or lyophilized mRNA-LNPs.

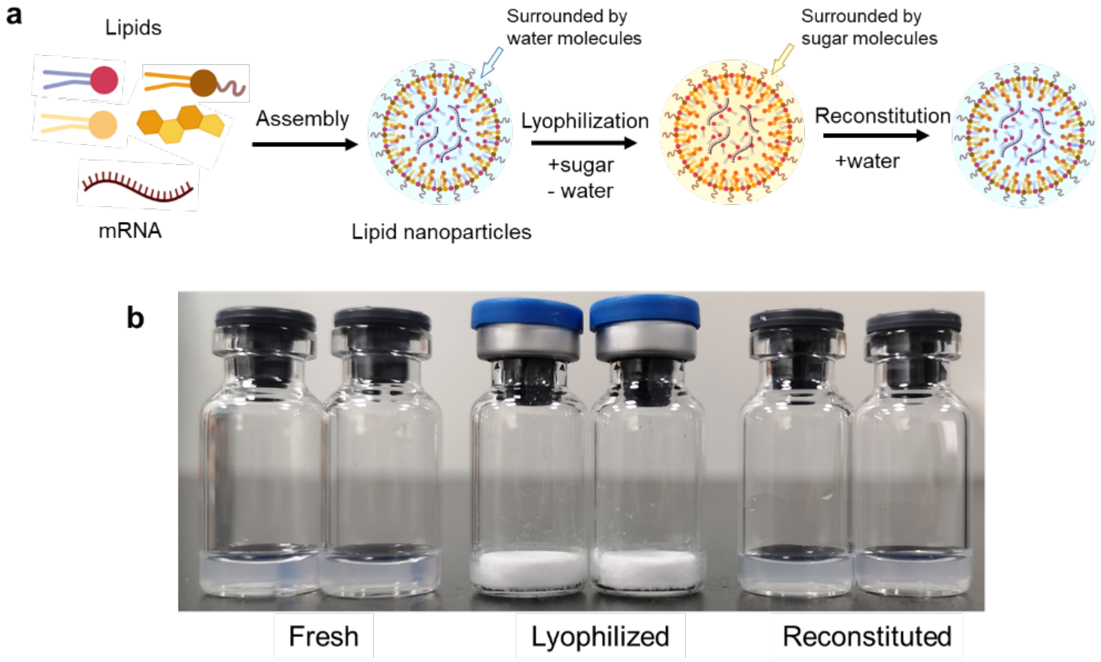

Supplementary Fig. S2|(a) Schematic diagram of mRNA-LNP preparation, lyophilization and reconstitution. (b) Photos of fresh mRNA-LNPs, lyophilized mRNA-LNPs and the reconstituted solutions.

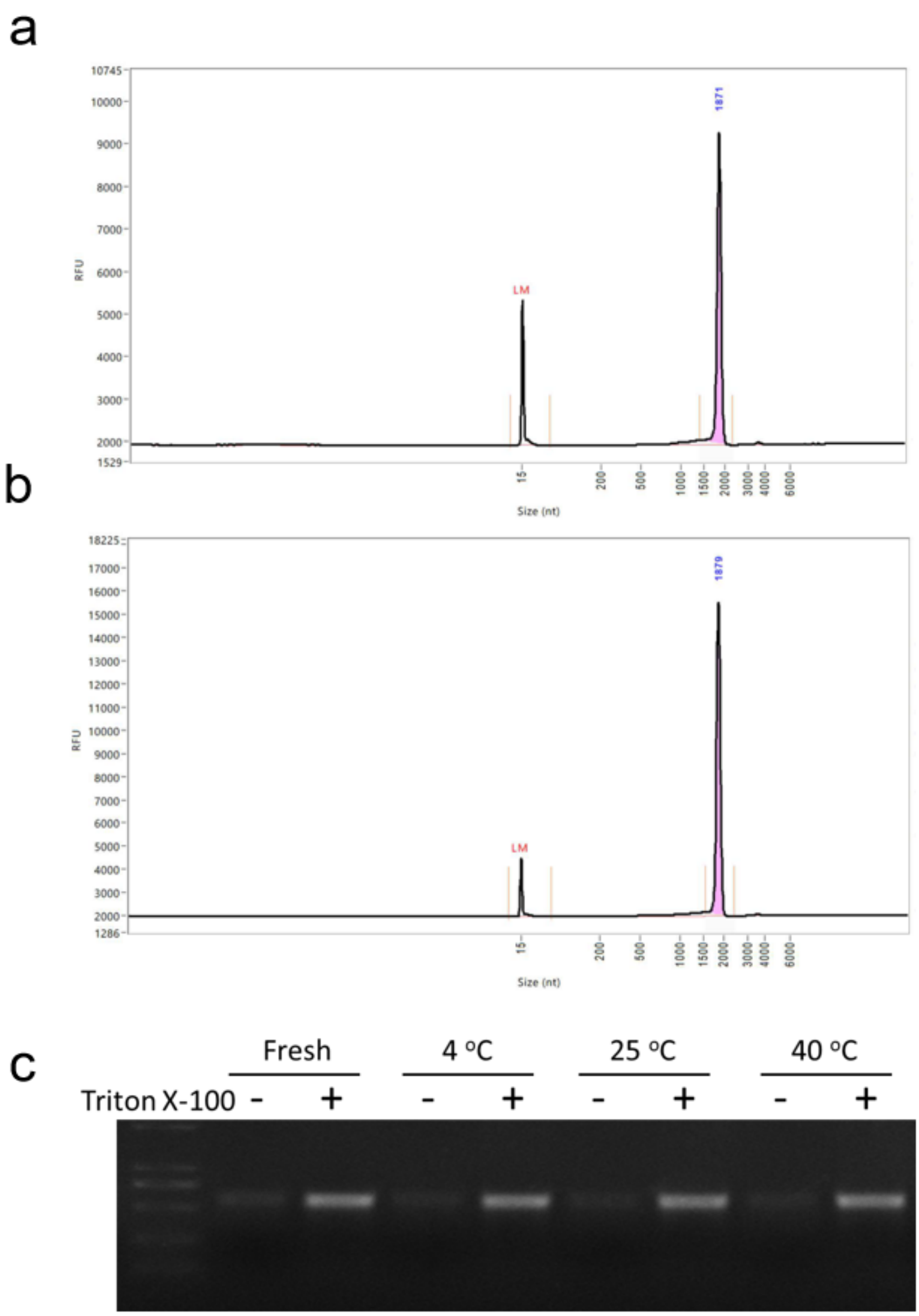

Supplementary Fig. S3|Microfluidic capillary electrophoresis analysis of (a) Omicron mRNA and (b) reconstituted LyomRNA-Omicron. The mRNA integrity was 94.1% and 93.9%, respectively. (c) Gel electrophoresis images of LyomRNA-Omicron LNPs after incubation at 4, 25, or 40°C for 10 days.

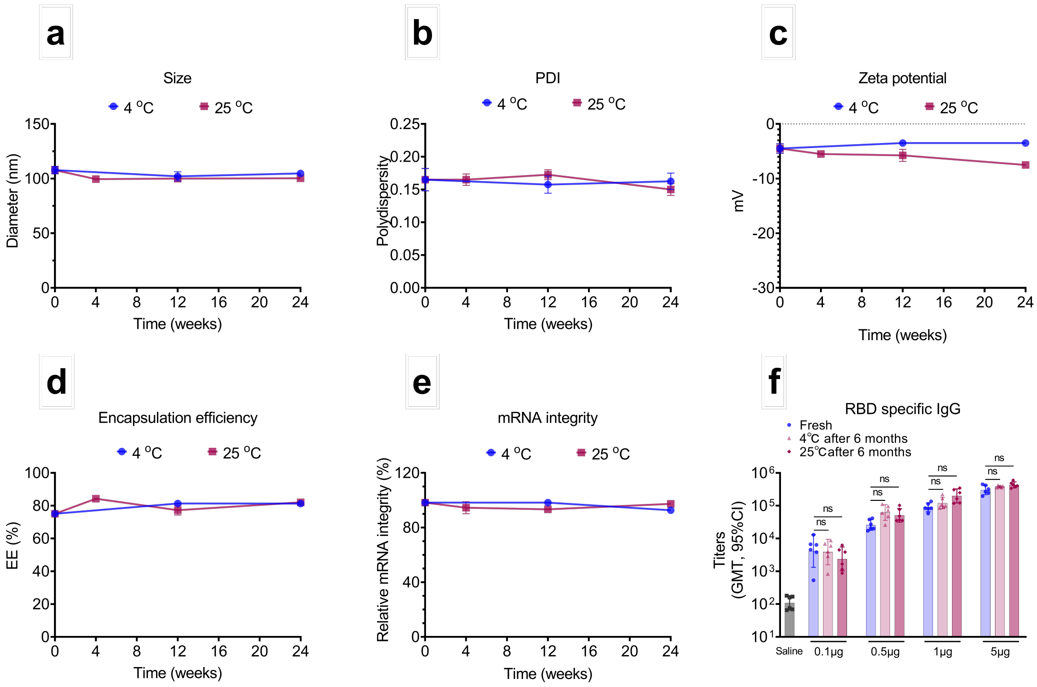

Supplementary Fig. S4| Long-term stability of freeze-dried mRNA-LNPs. Changes in the (a) size, (b) PDI, (c) zeta potential, (d) encapsulation efficiency and (e) mRNA integrity of LyomRNA-Omicron LNPs after incubation at 4 °C or 25°C for 6 months. (f) Total IgG titer of mice after one shot with freshly prepared mRNA-Omicron LNPs or LyomRNA-Omicron LNPs. Mice (n=6) were immunized with fresh or lyophilized mRNA-LNPs containing 0.1μg, 0.5μg, 1μg or 5μg mRNA at Day 0. Blood was collected and analyzed at Day 14. Data are presented as the mean with SEM (a-e) or the geometric mean ± 95% confidence interval (f). (f) statistical analyses were made by Kruskal-Wallis ANOVA with Dunn's multiple comparisons test. ns=not significant.

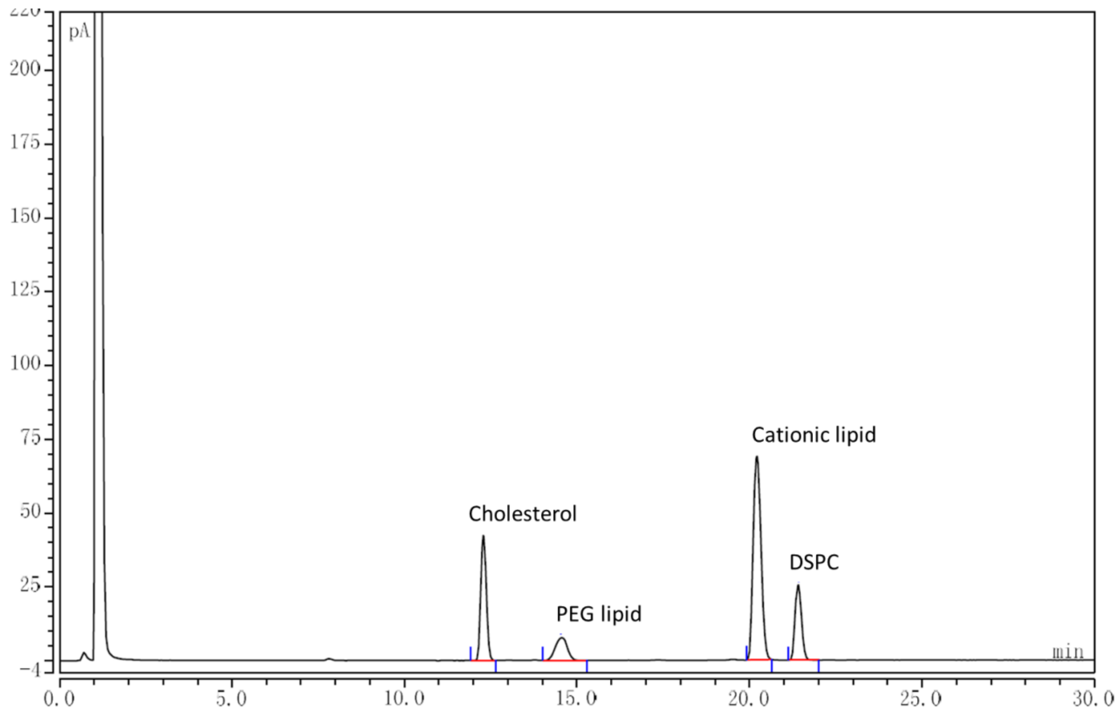

Supplementary Fig. S5| High chemical stability of LyomRNA-Omicron LNPs. LyomRNA-Omicron LNPs were incubated at 25°C for 6 months, and then the lipid components were analyzed with HPLC-CAD.

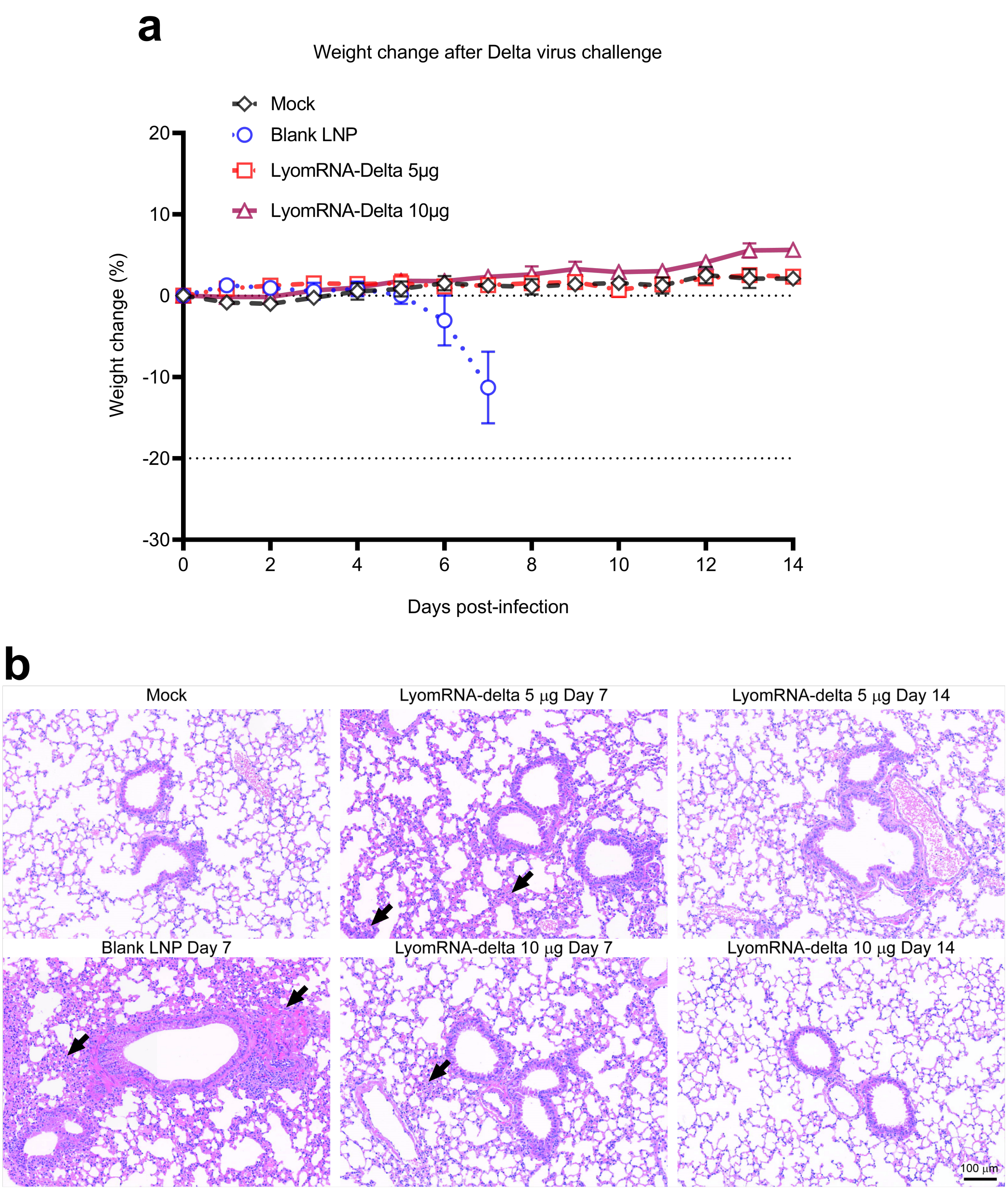

Supplementary Fig. S6| LyomRNA-Delta vaccine protect mice from upper and lower airway SARS-CoV-2 infection in the Delta virus challenge experiment. (a) Changes in body weight of mice post virus challenge. (b) Haematoxylin and eosin-stained lung sections were examined on day 7 and day 14 after the challenge. Data are presented as the mean with SEM. Mice immunized at week 0 and 3 with 5 or 10 μg of LyomRNA-Delta or blank LNP, were challenged with SARS-CoV-2 2 weeks post the 2nd immunization. 7 or 14 days post-challenge, mouse lungs were harvested to measure viral titers. At days 7 and 14 post-challenge, Haematoxylin and eosinstained lung sections were examined, and representative photomicrographs from each group. 7 days post challenge, lungs from blank LNP mice demonstrated diffuse alveolar damage characterized by thickening of the alveolar septa, severe inflammation (predominantly neutrophilic infiltration) presented within and surrounding small bronchioles

(arrowheads), and engorged alveolar capillaries that expanded due to infiltrating inflammatory cells. In the 5 μg of LyomRNA-Delta group, there was mild to moderate patchy expansion of alveolar septa by mononuclear and polymorphonuclear cells (arrowheads). In the 10 μg of LyomRNA-Delta group, occasional areas had inflammation (predominantly neutrophilic infiltration) presented closely associated with small airways (bronchioles) and adjacent vasculature (arrowheads). At days 14, lungs from 5 or 10 μg of LyomRNA group mice exhibited minimal to absent inflammation.

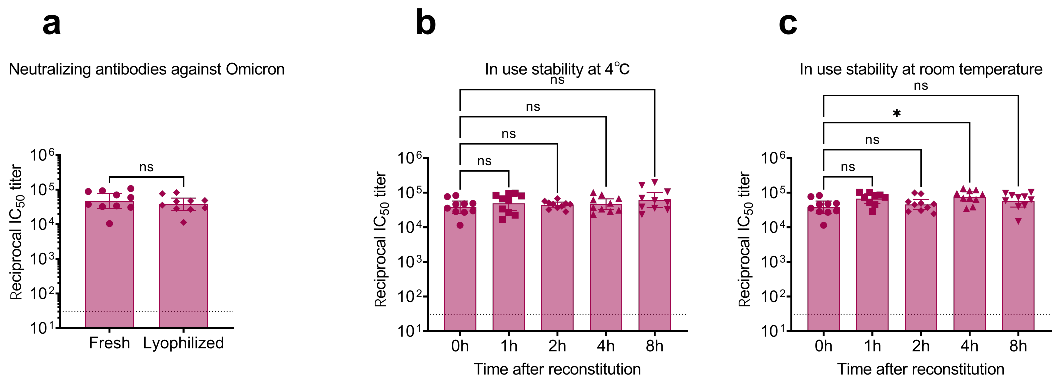

Supplementary Fig. S7|In-use stability of LyomRNA-Omicron. BALB/c mice (n=10) were immunized at a dose of 5 μg, with 2 shots administered at an interval of 21 days. Blood was acquired 14 days after the 2nd immunization for the detection of pseudovirus neutralizing antibodies. (a) Changes in immunogenicity after freeze drying. (b) Changes over time in immunogenicity of reconstituted vaccine at 4°C. (c) Changes over time in immunogenicity of reconstituted vaccine at ambient temperature. (a) Groups were compared by a two-sided Mann–Whitney test. (b-c) Comparisons of time points were made by Kruskal–Wallis ANOVA with Dunn's multiple comparisons test. ns=not significant, * = $p < 0.05$. Data are presented as the geometric mean ± 95% confidence interval.

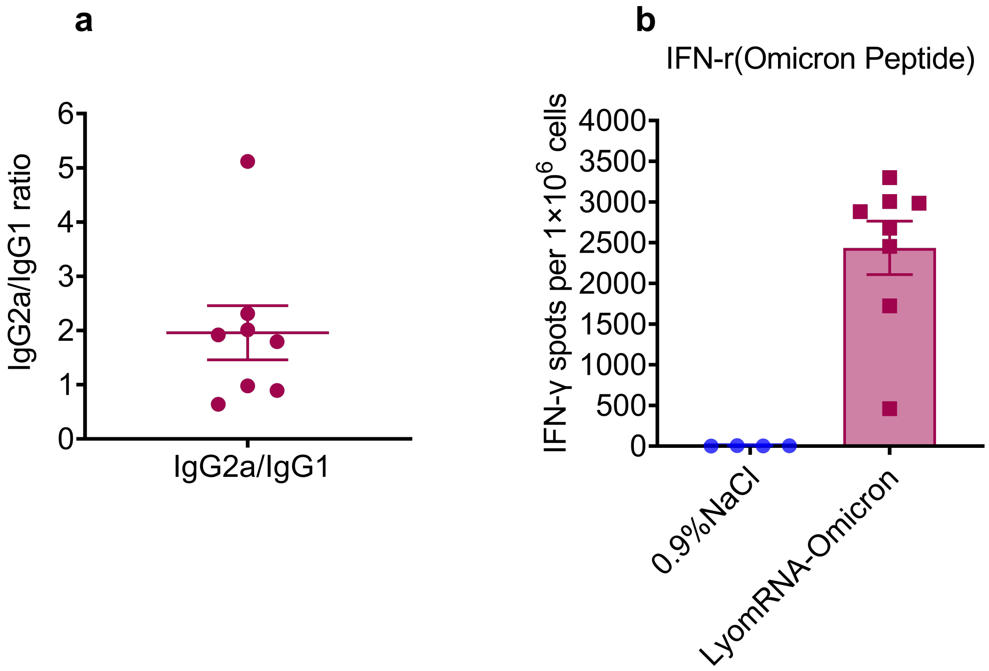

Supplementary Fig. S8|Mouse immunogenicity of LyomRNA-Omicron. (a) BALB/c mice (n=8) received two-dose immunization with 5 μg of LyomRNA-Omicron at an interval of 7 days. The ratio of plasma Omicron RBD-specific IgG2a/IgG1 was detected at day 14 after the last injection. (b) The number of IFN-r+ spots in the mouse spleen one month after the last dose was detected using ELISpot. Data are presented as the mean with SEM.

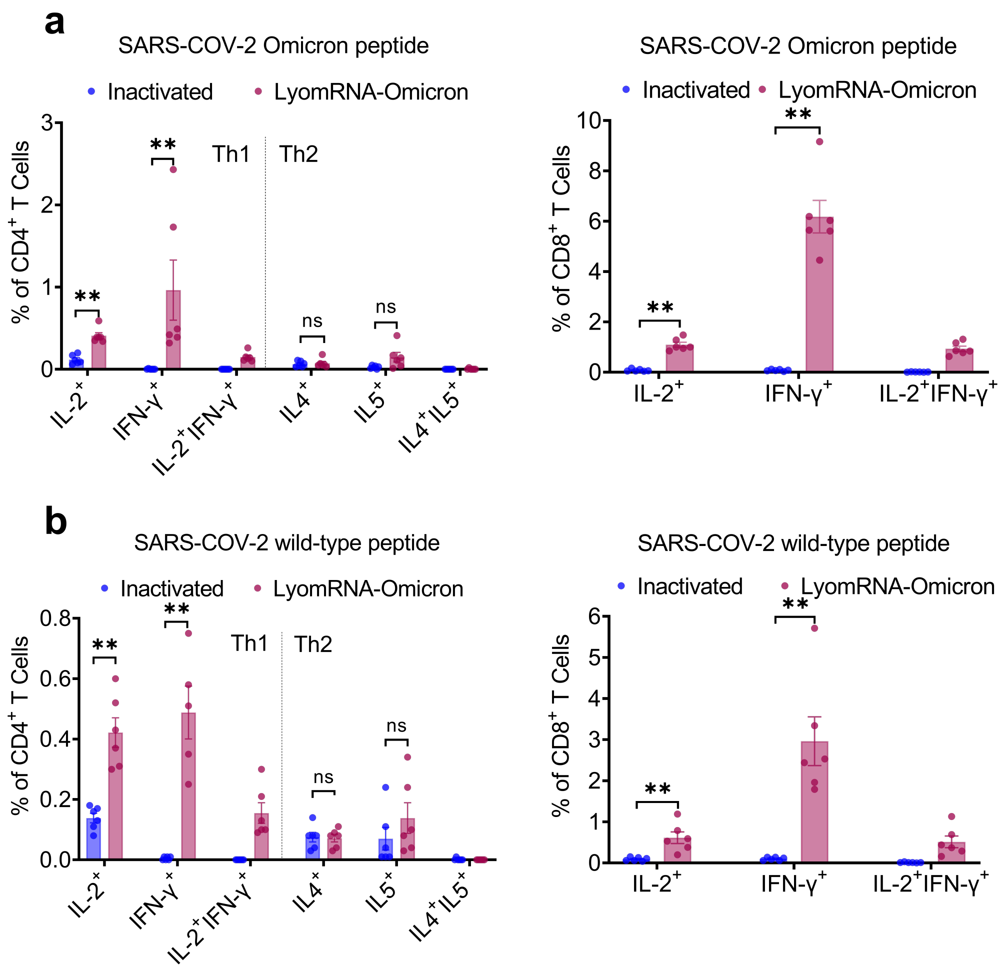

Supplementary Fig. S9|LyomRNA-Omicron induced a cellular immune response in BALB/c mice. BALB/c mice were vaccinated with LyomRNA-Omicron 5 μg or inactivated vaccine 0.65 U at an interval of 21 days. The spleens were obtained 28 days after the second shot for ICS. The frequencies of IL2/IFN-γ/IL4/IL5-positive CD4 T cells and IL2/IFN-γ-positive CD8 killer cells specific for SARS-CoV-2 Omicron NTD-RBD peptide (a) or wild-type NTD-RBD peptide (b) were detected. Vaccine groups were compared by a two-sided Mann–Whitney test. ns=not significant, ** = $p < 0.01$. Data are presented as the mean with SEM.

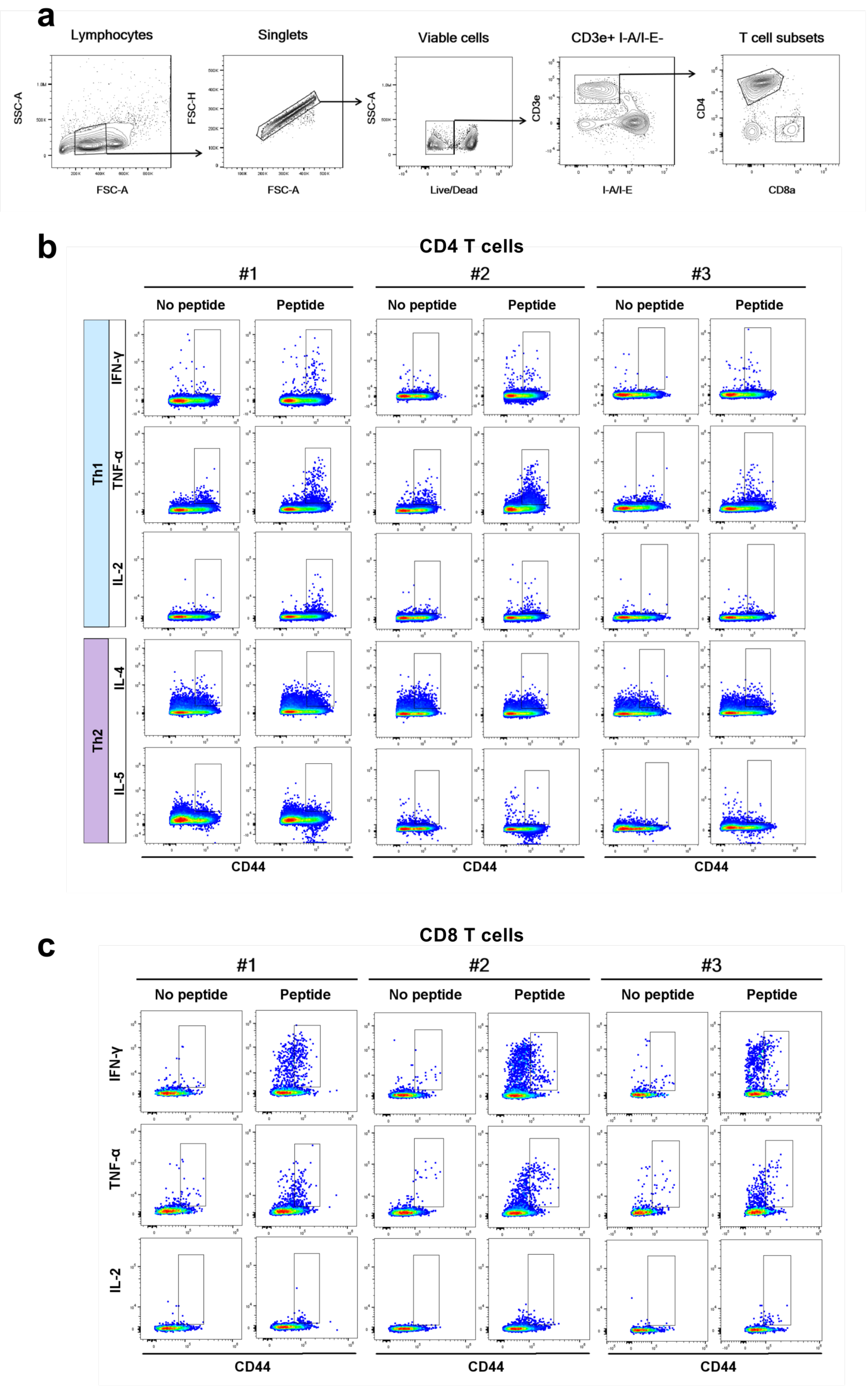

Supplementary Fig. S10| Flow cytometry panel to quantify Omicron NTD-RBD peptide specific T cells in BALB/c mice. (a) Hierarchical gating strategy to identify single, viable $CD4^{+}$ and $CD8^{+}$ T cells. (b-c) Gating summary of Omicron NTD-RBD

peptide-specific cytokine$^+$CD44hi$^+$ CD4$^+$ (b) and cytokine$^+$CD44$^+$CD8$^+$ (c) T cells elicited by 5 μg of LyomRNA-Omicron. #1/#2/#3 represent individual mice.

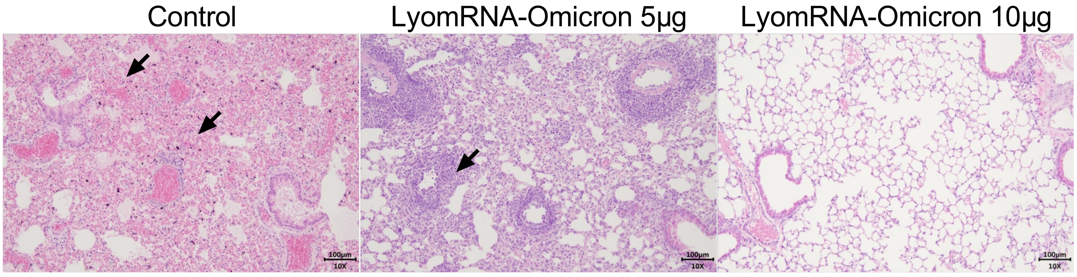

Supplementary Fig. S11| K18-hACE2 KI transgenic mice were immunized at a dose of 5 μg on days 0, 21, and 61, and were lethally challenged on day 73 with Omicron-BA.1. Pathological sections were evaluated 7 days after the challenge. 7 days post challenge, lungs from control mice demonstrated diffuse alveolar damage characterized by thickening of the alveolar septa, severe inflammation (predominantly neutrophilic infiltration) presented within and surrounding small bronchioles (arrowheads), and engorged alveolar capillaries that expanded due to infiltrating inflammatory cells. In the 5 μg of LyomRNA-Omicron group, there was moderate patchy expansion of alveolar septa by mononuclear and polymorphonuclear cells (arrowheads). Lungs from 10 μg of LyomRNA group mice exhibited minimal to absent inflammation.

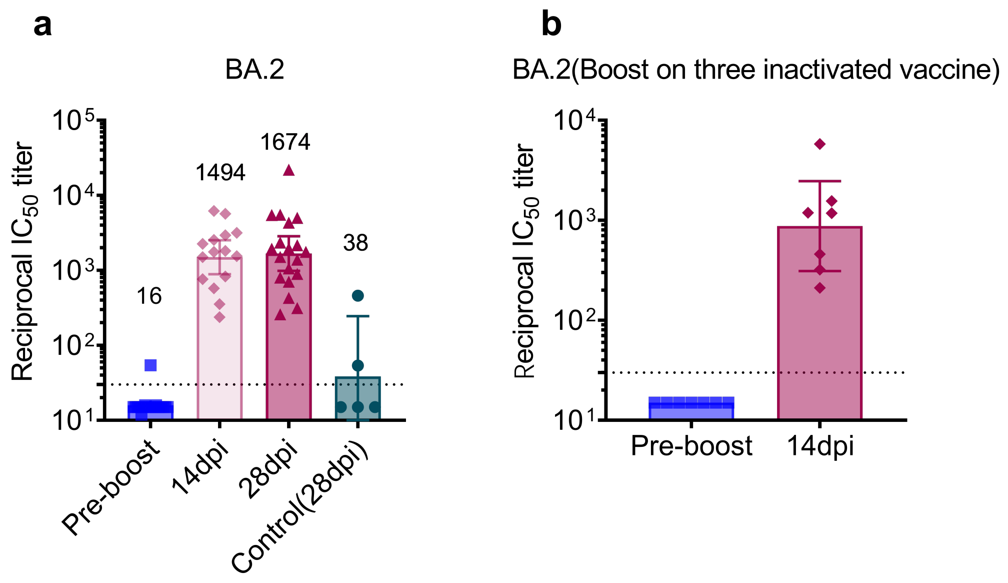

Supplementary Fig. S12| Plasma neutralizing antibodies against Omicron-BA.2 pseudoviruses after booster immunization with LyomRNA-Omicron. Omicron-BA.2 VSV pseudoviruses from a laboratory different from those in Figure 5 were used for detection of the titers of serum neutralizing antibodies. (a) Heterogeneous booster immunization with LyomRNA-Omicron (50 μg) following two doses of inactivated vaccine. (b) Heterogeneous booster immunization with LyomRNA-Omicron (50 μg) following three doses of inactivated vaccine. Data are presented as the geometric mean ± 95% confidence interval. The horizontal dashed line marks the LLOD. Values below the LLOD are set to 1/2 LLOD.

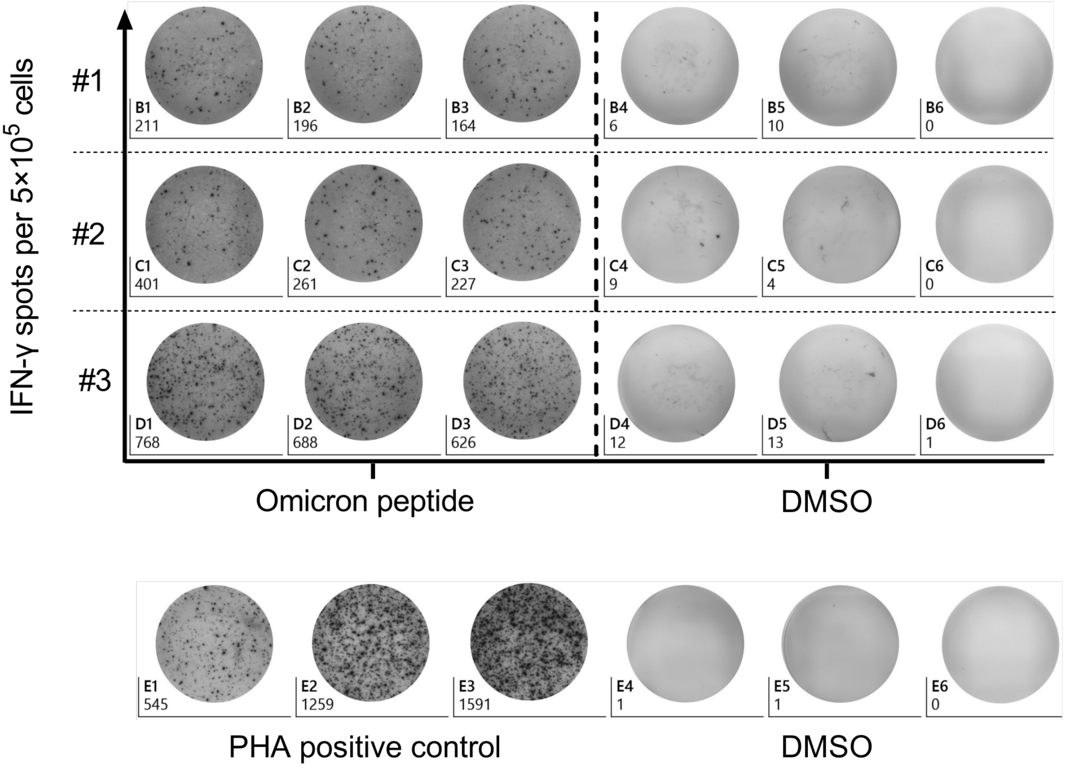

Supplementary Fig. S13| Cellular immune responses in humans after LyomRNA-Omicron booster immunization. #1/#/2/#3 represent 3 individual volunteers. A booster shot was given with 50 μg of LyomRNA-Omicron after two doses of inactivated vaccine. PBMCs (5×10^5 cells per well) were collected 59 days, 58 days and 21 days post-boost for #1, #2 and #3, respectively. The number of IFN-r spots was detected by ELISpot. Omicron NTD-RBD Omicron peptide or DMSO was used for stimulation in #1/#2/#3, and 5×10^5 cells per well were used in the PHA group.

Supplementary Table S1. Main physical characteristics of mRNA-LNPs before and after lyophilization

Samples	Fresh			Lyophilized		
	Size (nm)	PDI	EE (%)	Size (nm)	PDI	EE (%)
mRNA-Luc LNPs	74.1	0.19	94.7	93.9	0.15	85.6
mRNA-WT LNPs	92.4	0.14	92.1	117.7	0.16	87.0
mRNA-Delta LNPs	72.8	0.03	93.2	96.0	0.10	85.6
mRNA-Omicron LNPs	77.6	0.09	94.3	88.4	0.16	86.2

Supplementary Table S2. Vaccination history information of volunteers

Cohort Ⅰ：volunteers had received 2 doses inactivated vaccines (SARS-CoV-2 wild-type) historically			
**Number**	**Sex**	**age**	**days after the last shot**
**#1**	Male	23	D199
**#2**	Male	24	D243
**#3**	Male	24	D309
**#4**	Female	27	D257
**#5**	Male	30	D242
**#6**	Male	30	D287
**#7**	Male	32	D289
**#8**	Male	32	D317
**#9**	Male	33	D182
**#10**	Male	34	D404
**#11**	Male	34	D298
**#12**	Male	36	D295
**#13**	Male	36	D248
**#14**	Female	37	D183
**#15**	Male	37	D331
**#16**	Male	39	D280
**#17**	Male	43	D240
**#18**	Female	44	D293
**#19**	Male	49	D207
**Cohort Ⅱ：volunteers had received 3 doses inactivated vaccines (SARS-CoV-2 wild-type) historically**			
**Number**	**Sex**	**age**	**days after the last shot**
**#20**	Female	27	D110
**#21**	Female	42	D97
**#22**	Male	47	D111
**#23**	Male	59	D128
**#24**	Female	48	D118
**#25**	Male	40	D142
**#26**	Male	36	D134
